# Exploring microbial diversity in Greenland Ice Sheet supraglacial habitats through culturing-dependent and -independent approaches

**DOI:** 10.1093/femsec/fiad119

**Published:** 2023-10-03

**Authors:** Ate H Jaarsma, Katie Sipes, Athanasios Zervas, Francisco Campuzano Jiménez, Lea Ellegaard-Jensen, Mariane S Thøgersen, Peter Stougaard, Liane G Benning, Martyn Tranter, Alexandre M Anesio

**Affiliations:** Department of Environmental Science, Aarhus University, Frederiksborgvej 399, 4000 Roskilde, Denmark; Department of Environmental Science, Aarhus University, Frederiksborgvej 399, 4000 Roskilde, Denmark; Department of Environmental Science, Aarhus University, Frederiksborgvej 399, 4000 Roskilde, Denmark; Department of Environmental Science, Aarhus University, Frederiksborgvej 399, 4000 Roskilde, Denmark; Department of Environmental Science, Aarhus University, Frederiksborgvej 399, 4000 Roskilde, Denmark; Department of Environmental Science, Aarhus University, Frederiksborgvej 399, 4000 Roskilde, Denmark; Department of Environmental Science, Aarhus University, Frederiksborgvej 399, 4000 Roskilde, Denmark; German Research Centre for Geosciences, Helmholtz Centre Potsdam, Telegrafenberg, 14473 Potsdam, Germany; Department of Earth Sciences, Freie Universität Berlin, Malteserstr. 74-100, 12249 Berlin, Germany; Department of Environmental Science, Aarhus University, Frederiksborgvej 399, 4000 Roskilde, Denmark; Department of Environmental Science, Aarhus University, Frederiksborgvej 399, 4000 Roskilde, Denmark

**Keywords:** amplicons, Greenland Ice Sheet, *in situ* culturing, isolates, MAGs, metagenome

## Abstract

The microbiome of Greenland Ice Sheet supraglacial habitats is still underinvestigated, and as a result there is a lack of representative genomes from these environments. In this study, we investigated the supraglacial microbiome through a combination of culturing-dependent and -independent approaches. We explored ice, cryoconite, biofilm, and snow biodiversity to answer: (1) how microbial diversity differs between supraglacial habitats, (2) if obtained bacterial genomes reflect dominant community members, and (3) how culturing versus high throughput sequencing changes our observations of microbial diversity in supraglacial habitats. Genomes acquired through metagenomic sequencing (133 high-quality MAGs) and whole genome sequencing (73 bacterial isolates) were compared to the metagenome assemblies to investigate abundance within the total environmental DNA. Isolates obtained in this study were not dominant taxa in the habitat they were sampled from, in contrast to the obtained MAGs. We demonstrate here the advantages of using metagenome SSU rRNA genes to reflect whole-community diversity. Additionally, we demonstrate a proof-of-concept of the application of *in situ* culturing in a supraglacial setting.

## Teaser

Various culturing-dependent and -independent methods were used to explore microbial diversity on the Greenland Ice Sheet, highlighting the benefits of combining different approaches to capture genomic diversity.

## Introduction

The Earth primarily consists of environments that remain cold (< 5°C) throughout the year, which contain communities of microorganisms adapted to these conditions (Margesin and Collins 2019). In recent years, the microbiome of this cryosphere is becoming better described (Bourquin et al. 2022). It is now widely accepted that glaciers and ice sheets are biomes that are largely microbially driven, containing niches for microbes adapted to cold temperatures, high UV radiation, freeze–thaw cycles, and long, dark winters. These ecosystems are known to harbor a diverse range of microbes, including bacteria, fungi, eukaryotic algae, and viruses (Anesio et al. 2017), but are still widely understudied (Edwards et al. 2020). Researching the diversity of microbes in the cryosphere is necessary not only to catalog the endemic species of an environment, but also to understand how these organisms may interact with each other and influence their own and adjacent habitats. For example, microbes from the supraglacial environment influence the biogeochemistry of downstream regions as they are transported off the ice sheet during the melt season (Stevens et al. 2022).

The Greenland Ice Sheet, being the northern hemisphere’s largest body of ice (Stibal et al. 2015), is an important subject of study in this regard. Several studies described the microbial communities of supraglacial environments on the Greenland Ice Sheet, such as ice and cryoconite holes (Stibal et al. 2015, Hauptmann et al. 2017, Perini et al. 2019, Mogrovejo et al. 2020, Poniecka et al. 2020, Lutz and Bradley 2021, Millar et al. 2021). On the ice surface, dark-pigmented eukaryotic algae are the main primary producers (Anesio et al. 2017). The ice surface is darkening due to blooms of these pigmented algae, leading to increasing glacial melt off (Cook et al. 2020). Cyanobacteria are the main primary producers in cryoconite holes (Anesio and Laybourn-Parry 2012), which are cylindrical holes filled with a layer of granular sediment that is a mix of biological and mineral material (Cook et al. 2016). Heterotrophic bacteria are also common in these supraglacial habitats (Lutz et al. 2017). Alpha- and Beta-Proteobacteria, Bacteroidetes, and Actinobacteria are commonly reported in these environments (Stibal et al. 2015, Hauptmann et al. 2017, Perini et al. 2019, Mogrovejo et al. 2020, Poniecka et al. 2020, Lutz and Bradley 2021, Millar et al. 2021).

Most microbes are difficult to culture *in vitro*, and therefore the study of their diversity, physiology, and metabolism proves challenging under controlled conditions. However, culture-independent approaches can be utilized to circumvent this. In recent years, High-Throughput Sequencing technologies (Reuter et al. 2015) have made it cheaper and easier to access genomic information from environmental microorganisms. Metagenome sequencing (MGS) can be used to obtain metagenome-assembled genomes (MAGs). This enables the study of the functional potential of microbes without the need of having the organisms in culture. There are, however, still benefits of having microbes in culture, especially if investigating phenotypes and metabolic capabilities under controlled conditions is the goal (Poniecka et al. 2020). In order to target this uncultured “microbial dark matter,” novel culturing methods have been developed in recent years (Lewis et al. 2020). One such method is *in situ* incubation, which involves physically separating microbial cells in small culture chambers and incubating them in the same environment that the sample was collected from (Kaeberlein et al. 2002, Jung et al. 2016, Liu et al. 2020). This allows the diffusion of growth factors from outside into the chambers through a semipermeable membrane. An example of this is the “isolation chip” or *ichip*, which is an array of separated culture chambers, made by using a plate of metal or plastic with agar-filled holes (Berdy et al. 2017). These are inoculated with diluted sample and closed off on both sides using a membrane. This allows the chip to be placed back into the original environment for culturing before being transported to the laboratory. Interestingly, it is found that each round of incubation *in situ* adapts the isolated strains further for growth under laboratory conditions through a domestication process. A process in which isolates are first grown in their natural environment, therefore, results in the isolation of more strains than using conventional techniques only (Berdy et al. 2017). Application of the *ichip* has successfully led to the discovery of a new antibiotic, teixobactin (Ling et al. 2015). The *ichip* concept has been applied in permafrost (Marcolefas et al. 2019) and ice wedge soil (Goordial et al. 2017). To our knowledge, however, it has never been applied in a supraglacial environment.

Several studies have previously investigated the microbial communities of supraglacial habitats of the Greenland Ice Sheet through either culturing or culturing-independent approaches, or both (Musilova et al. 2015, Stibal et al. 2015, Cameron et al. 2016, Hauptmann et al. 2017, Perini et al. 2019, Mogrovejo et al. 2020, Poniecka et al. 2020, Millar et al. 2021). Most papers, however, do not focus on comparing cultured diversity to diversity that is observed through sequencing, as was for instance done for Svalbard permafrost (Dziurzynski et al. 2022, Sipes et al. 2022) and Tibetan glaciers (Liu et al. 2022).

In this study, the microbial diversity of supraglacial habitats of the Greenland Ice Sheet was assessed through a combination of culturing and culturing-independent approaches. We demonstrate the power of MGS to visualize relative abundances of small subunit (SSU) rRNA genes of the entire microbial community. Considering the scarcity of genomes reported from the Greenland Ice Sheet, we also investigate which part of the community present in these habitats is discoverable either as a MAG, through MGS, or as an isolate in axenic culture. As part of our culturing approach, a novel *in situ* culturing method (here referred to as culture chips and culture chambers) was employed for the first time on the Greenland Ice Sheet.

## Materials and methods

### Sample collection

**Figure 1.**
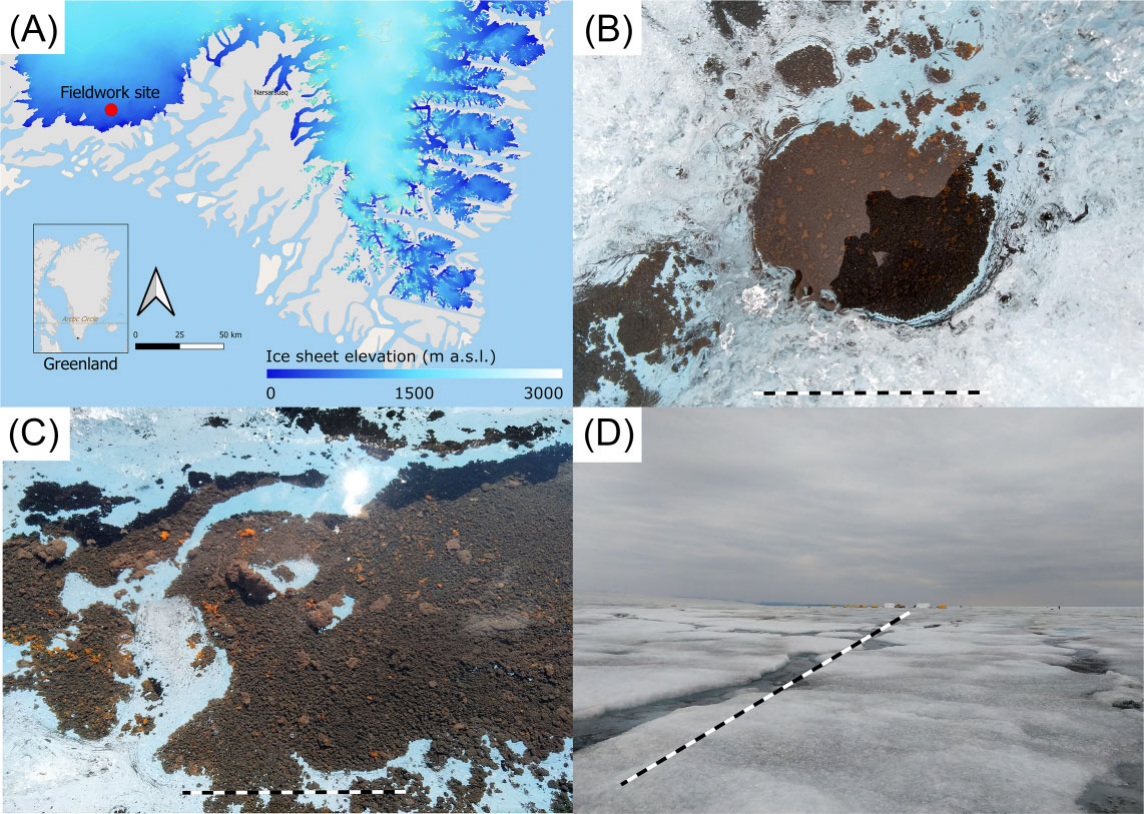
(A) Fieldwork site on southwest margin of Greenland Ice Sheet. OpenStreetMap and ArcticDEM (Porter et al. 2023) were used to create map layers. (B)–(D) examples of supraglacial habitats investigated in this study; cryoconite (B, scale bar = 20 cm), biofilm in cryoconite hole (C, scale bar = 10 cm), and dark ice surface (D, scale bar =∼ 120 m).

Sample collection and *in situ* incubation were done in July–August 2021 during the Deep Purple (https://www.deeppurple-ercsyg.eu/) fieldwork campaign. The campsite was on the ice sheet in the south of Greenland, about 7.5 km from the margin, 61.10138895, −46.8481389, 617 m a.s.l. (Fig. 1A, map generated using OpenStreetMap and ArcticDEM; Porter et al. 2023).

Environmental samples of the dark ice surface and cryoconite holes were taken across a 100 m × 100 m area (Fig. 1B–D). Ice samples were taken by scraping approximately two vertical cm of the dark ice surface with a field-sterilized ice axe (cleaned with 70% ethanol, and conditioned with similar ice) and stored in sterile 4 l Whirl-pak bags to melt at ambient temperature of 5–10°C. Cryoconite sediment was collected with a polycarbonate aquarium pipette from 30 different cryoconite holes with various ways of hydrological connectivity within the aforementioned area. A total of 18 kg of surface ice and 3.5 kg of cryoconite sediment were collected. Each environmental sample was combined and homogenized in their own sterile sample bags. Subsamples of both the ice and cryoconite samples were subsequently taken in a 50 ml tube and kept cool during transport back to the lab. In total, three 4 ml cryotubes were filled with cryoconite sediment for DNA extraction. Biomass for DNA extraction from the ice sample was collected by filtering three technical replicates of 500 ml melted ice onto Sartorius cellulose nitrate filters (0.2 µm). Filters were rolled up and stored in 4 ml cryotubes. Two additional samples were a viscous suspended biofilm from a cryoconite hole and a red snow sample. Both were collected in 50 ml tubes and kept cool during transport back to the lab. A subsample of the biofilm was taken for DNA extraction. All samples for DNA extraction (cryoconite, ice, and biofilm) were frozen in the field camp and kept at −20°C through transport to Aarhus University, Roskilde, Denmark.

### Culture chips and chambers

#### Design

**Figure 2.**
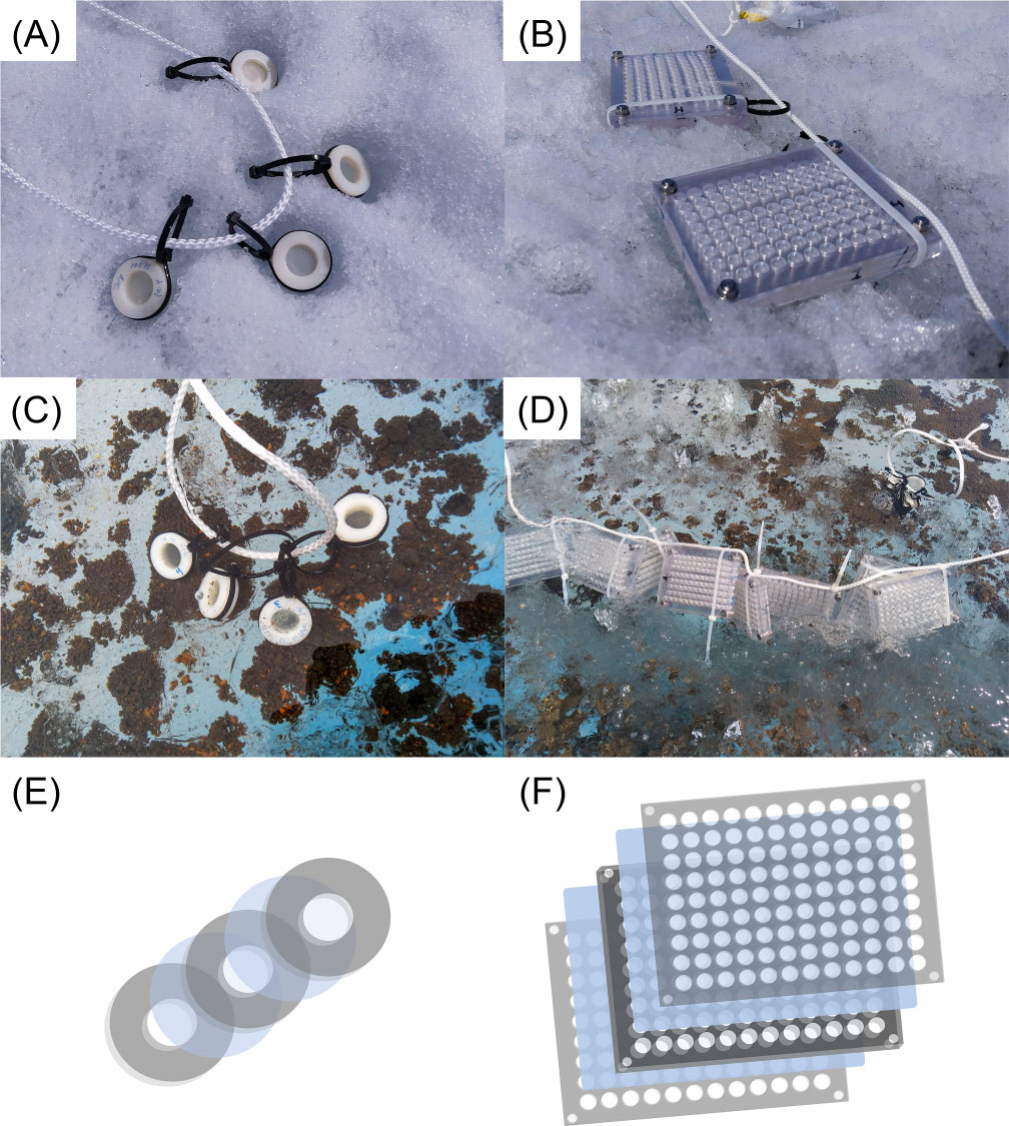
Culture chips (B) and (D) and chambers (A) and (C) deployed on ice (A) and (B) and submerged in cryoconite hole (C) and (D). Design of culture chambers (E) and culture chips (F) is based on a middle layer to form the chamber, which is closed off by two semipermeable polycarbonate membranes, here shown in blue. Two additional plastic outer layers protect the assembly.

The culture chips used for *in situ* culturing were inspired by an existing protocol (Berdy et al. 2017) with minor alterations. The 1-cm thick plate was made from polycarbonate and had an array of 96 holes that could be filled with solid medium. The 3-mm thin outer layers serve to protect the semipermeable membrane. The plastic layers were sterilized by autoclaving prior to use in the field. This stack was bolted down to stay together (Fig. 2B, D, and F). The design of the culture chambers was similar but made with three autoclaved plastic washers that formed one bigger growth chamber when stacked together with membranes in between (Fig. 2A, C, and E).

#### *In situ* deployment

Culture chips and chambers were first half-assembled by gluing polycarbonate hydrophilic membranes (Cytiva Nuclepore, USA; 0.03 µm) to one side of the middle layer using aquarium silicone glue, applied to the plastic in a thin layer.

To prepare the inoculum, the homogenized cryoconite sediment and ice surface samples were serially diluted with *in situ* autoclaved (heated in pressure cooker to >117°C for 20 min) glacial stream water. Cryoconite sediment sample dilutions used were 10^−3^–10^−6^, and 10^−1^–10^−4^ for ice; based on previously reported cell counts for both environments (Nicholes et al. 2019). A total of two culture chips were made for each of the four dilutions. A volume of 400 µl of the dilutions were added to 50 ml tubes, in duplicate, for both the ice and cryoconite inocula. To one series of four dilutions for both cryoconite and ice samples, 500 µl nystatin ready-made solution (Sigma Aldrich, USA) was added. In addition, 40 ml Reasoner’s 2 agar (R2A) (Linde et al. 2000) (Alpha Biosciences), that was supplemented with 18% glycerol to serve as a freezing point depressant in the solid medium, because of concerns for the agar gel deteriorating after freeze–thaw cycles on the ice, was added to each tube. After mixing, this medium was used to fill all wells of the culture chips, except for the last column of eight wells on each chip, which was filled with R2A+ 18% glycerol without inoculum, as a negative control. A few mm headspace was left on top of each well. After the agar was solidified, another membrane was applied to the still open side, sealing the chip. The outer layers were then bolted on to finish the assembly.

For the culture chambers, 10 µl of the 10^−4^ dilution of the cryoconite sample, or the undiluted ice sample, were added in triplicate to 2 ml tubes. A volume of 12.5 µl nystatin ready-made solution was added to two of these tubes. To one of the tubes with nystatin, 2 µl streptomycin (50 mg ml^−1^) was also added. This way, both the ice and cryoconite inoculum was mixed with nystatin, streptomycin + nystatin, or no antibiotics. One ml R2A + 18% glycerol agar was added to each tube, this was used to fill the already half-assembled culture chambers. A negative control chamber with nonamended sterile agar was also made.

Once assembled, the culture chips and chambers were left to incubate *in situ* (Fig. 2), inside a large cryoconite hole in which the chips and chambers containing the cryoconite inoculum were submerged (Fig. 2C and D). The chips and chambers made with ice inoculum were deposited on a patch of dark ice for incubation (Fig. 2A and B). The cryoconite and ice culture chips were left to incubate for 18 and 16 days, respectively. The cryoconite and ice culture chambers were left for 17 and 15 days, respectively. After this time, the *in situ* culturing devices were removed from the environment and placed in sterile Whirl-pak bags, covered in sterile glacial stream water and stored at about 5°C during transportation back to the home laboratory.

### Plates

A volume of 100 µl of cryoconite sediment and dark ice were plated onto R2A + 18% glycerol agar, supplemented with either 100 µg ml^−1^ streptomycin, 12.5 µl nystatin, both, or no antibiotics. A volume of 100 µl of the snow and the biofilm samples were also plated on R2A + 18% glycerol agar plates without antibiotics. The agar plates were incubated at ambient temperature in the lab tent (∼ 5°C) in parallel with the *in situ* culturing and at the end of the inoculation they were transported back to Aarhus University, Roskilde, Denmark at 5°C. Back in the lab, the four environmental samples (100 µl) were plated onto R2A agar in triplicate, and incubated at 5°C, 10°C, and room temperature (20°C).

### Culturing of isolates

Data from the nearby PROMICE (Fausto et al. 2021) revealed that during the *in situ* incubation (17 July 2021–4 August 2021) the air temperatures ranged between 1.15 and 8.51°C, with an average of 3.70 +/− 1.38°C. Full weather station data can be found in the Supplementary Information S1. Isolates were primarily cultured at 5°C to stay close to this range.

After returning to the lab, the agar from the culture chambers was extruded and spread on R2A plates. Culture chips were first dried, opened, and examined under a dissection microscope. Each well was inspected, and microbial growth was streaked onto R2A plates with a sterile toothpick. Colonies were also observed on the outside of the membrane, and some were streaked onto R2A.

All plates were subsequently incubated at 5°C until colonies appeared (between 2 weeks and 2 months). Colonies were restreaked onto new plates, selected based on different morphologies, until visible axenic cultures were obtained. Isolates picked from plates that initially were incubated at 10°C or room temperature were eventually also incubated at 5°C for following rounds of culturing.

### Sequencing of isolates and environmental samples

#### Sanger sequencing of isolates

Axenic cultures were grown in liquid culture (R2B, Alpha Biosciences, USA) at 5°C on an orbital shaker (200 r m^−1^) until opaque (between a few days to 2 weeks), and subsequently transferred to cryotubes creating a stock in 18% glycerol, flash frozen and stored at −80°C. DNA was extracted by resuspending the pellet of 1 ml overnight culture in 100 µl milliQ water and boiling the suspension for 10 min. These extracts were stored at −20°C. The V1–V8 region of the 16S (for bacteria) and the 5′ terminal domain of the LSU rRNA genes (for yeasts) were targeted for amplification. The following primers were used for prokaryotes 16S rRNA gene 27f (AGA GTT TGA TCM TGG CTC AG), 16S 1392r (ACG GGC GGT GTG TGT RC), and for eukaryotes (yeast) 26S rRNA gene LSU NL1 forward (GCA TAT CAA GCG GAG GAA AAG), LSU NL4 reverse (GGT CCG TGT TTC AAC ACG G). PCR master mix contained 12.5 µl 2x Ultramix (PCR Biosystems, London, England) 0.5 µl of both primers (10 µM) and BSA (10 mg ml^−1^), 9 µl H_2_O, 2 µl template DNA. PCR protocol for 16S was as follows: 95°C for 2 min, 30 cycles with 95°C for 15 s, 55°C for 15 s, 72°C for 40 s, and final elongation at 72°C for 4 min. LSU protocol was the same but using 45 s for denaturation, 30 s for annealing at 54°C, and 2 min elongation. A volume of 5 µl of PCR product, together with 5 µl of forward primer, was sent for Sanger sequencing through the EZ-Seq service at Macrogen Europe (Amsterdam, the Netherlands). Geneious Prime version 2022.2.2 (Biomatters, Auckland, New Zealand) was used to check the quality of sequencing results, trim ends, and BLAST the results against the NCBI 16S and 18S ribosomal RNA databases.

#### Whole genome sequencing of cultures

Glycerol stocks from −80°C were used to inoculate overnight cultures in 2 ml R2B. Overnight cultures of each isolate were incubated in duplicate at both room temperature and 5°C while shaking (200 r m^−1^), the fastest growing culture used for DNA extraction. DNA extraction was carried out using a Gentra Puregene kit (Qiagen, Hilden, Germany), according to the manufacturer’s protocol, except for the DNA hydration solution. A total of 10 mM Tris, 50 mM NaCl pH 8.0 was used instead. Nextera XT kit (Illumina, San Diego, USA) was used for library preparation. DNA was resuspended in PCR water instead of resuspension buffer. The pooled genomes were sequenced on a NextSeq 500 using the MID output flow cell and the v2.5, 300 cycle chemistry (Illumina), resulting in 39 gigabases total output passing the Q30 threshold. The raw reads were processed through our automated whole genome sequencing (WGS) pipeline (Campuzano 2023). Briefly, the raw reads were trimmed, assembled, annotated, and compared to each other. Statistics regarding the quality of the reads, the completion of the assemblies and so on, were also calculated. Full specifications of the programs used, including their versions and options are listed in the Github repository (https://github.com/AU-ENVS-Bioinformatics—https://zenodo.org/badge/latestdoi/546561474). For the genomes with minor contamination (< 3%), BBNorm (sourceforge.net/projects/bbmap) was used with the following options: target=200 min=30 t=50 tossbadreads=t. The decontaminated reads were run through the pipeline again.

#### Amplicon sequencing of environmental samples

DNA was extracted from three technical replicates of cryoconite sediment, filters with biomass from ice, and the biofilm using the DNeasy PowerLyzer Power Soil kit (Qiagen). The following universal prokaryotic 16S rRNA gene and eukaryotic 18S rRNA gene primers were used for amplicon library building. 16S forward 341F (TCGTCGGC AGCGTCAG ATGTGTAT AAGAGACA GCCTAYGG GRBGCASC AG) and reverse 806R (GTCTCGTG GGCTCGGA GATGTGTA TAAGAGAC AGGGACTA CNNGGGTA TCTA AT) (Hansen et al. 2012), and 18S forward 528F (TCGTCGGC AGCGTCAG ATGTGTAT AAGAGACA GGCGGTAA TTCCAGCT CCAA) and reverse 706R (GTCTCGTG GGCTCGGA GATGTGTA TAAGAGAC AGAATCCR AGAATTTC ACCTCT) (Cheung et al. 2010) all at 10 µM concentration. The amplicon library building was performed by a two-step PCR, as described by Feld et al. (2016) and Albers et al. (2018) with slight modifications. PCR reactions were conducted on a SimpliAmp Thermal Cycler (Applied Bio-systems, Waltham, USA). In each reaction of the first PCR, the mix contained 12.5 µl of 2x PCRBIO Ultra Mix (PCR Biosystems), 0.5 µl of forward and reverse primer, 0.5 µl of bovine serum albumin (BSA) to a final concentration of 0.025 mg ml^−1^, 6 µl of PCR-grade water, and 5 µl of template. The reaction mixture was preincubated at 95°C for 2 min, followed by 33 cycles of 95°C for 15 s, 55°C for 15 s, 72°C for 40 s, with a final extension performed at 72°C for 4 min. Samples were subsequently indexed by a second PCR. For this, amplification was performed in 28 µl reactions with 12.5 µl of 2x PCRBIO Ultra Mix (PCR Biosystems), 2 µl of indexing primers (P7/P5), 6.5 µl of PCR-grade water, and 5 µl of PCR1 product. The cycling conditions included initial denaturation at 98°C for 1 min, followed by 13 cycles of denaturation at 98°C for 10 s, annealing at 55°C for 20 s, and extension at 72°C for 40 s, with a final extension performed at 72°C for 5 min. The final PCR products were purified with 15 µl magnetic beads (MagBio Genomics, Gaithersburg, USA) according to the manufacturer’s instructions and eluted in 27 µl buffer. Electrophoresis 1% agarose gels and TapeStation D1000 DNA ScreenTape (Agilent, Santa Clara, USA) were run to check the quality of the libraries. Finally, the concentrations of the libraries were measured on a Qubit 4.0 fluorometer (Invitrogen, USA), and these were then equimolarly pooled. The final pooled libraries from 16S and 18S were sequenced on an Illumina MiSeq using the V2 chemistry (Illumina) resulting in 2 × 250 bp reads.

#### Metagenome sequencing

The extracted DNA from environmental samples was also used for shotgun MGS. Three replicates of dark ice and cryoconite sediments where used, but only one replicate of the biofilm was used for MGS. Ice and cryoconite samples were prioritized since they were more widely used in the culturing approach. Hence, in total seven libraries were prepared using the Ultra FS II DNA Library Prep Kit for Illumina (New England Biolabs, Ipswich, USA) following the manufacturer’s protocol. The libraries were pooled equimolarly, and the pool was run in a TapeStation 4150 to check for insert size distribution and the presence of adapter–primer dimers, using a D1000 DNA ScreenTape. Dilution and denaturation of the library was performed according to Illumina’s recommendations before sequencing on a NextSeq 500 using the high output flow cell, and the v2.5, 300 cycle chemistry. 140 gigabases passing the Q30 threshold were obtained.

### Metagenomic assembly, binning, and taxonomic identification

Metagenomic reads were processed with metaWRAP v = 1.3.2 (Uritskiy et al. 2018) as described in the tool’s github page Useage_tutorial.md (Accessed 10 October 2022). Briefly, single sample assembly was done with metaspades v3.11.1 (Nurk et al. 2017) on the seven samples [ice surface (*n* = 3), biofilm (*n* = 1), and cryoconite sediment (*n* = 3)]. Metagenomic bins were constructed with metabat2 v2.12.1 (Kang et al. 2019), maxbin2 v2.2.5 (Wu et al. 2016), and CONCOCT v 1.0.0 (Wehrmann et al. 2017). Resulting bins were reassembled and refined under a threshold of > 90% completeness and < 5% contamination, resulting in high quality assembled metagenomes. The contigs within each MAG were mapped back to all metagenome sample assemblies and read recruitment was calculated in “Reads per million,” which takes into account the number of mapped reads, the gene length, normalized to one million (Equation 1).


(1) \begin{eqnarray*}
&& {\mathrm{Reads}}\,\,{\mathrm{per}}\,\,{\mathrm{million}} \nonumber \\
&& = \left(\sum {\mathrm{mapped}}\,\,{\mathrm{contigs}}/\left( {{\mathrm{gene}}\,\,{\mathrm{length}}/1000} \right)\right)/1,\!000,\!000.
\end{eqnarray*}

All MAG taxonomy was identified using GTDB-Tk v 2.1.1 (Parks et al. 2018) and manually changed to match NCBI taxonomy at phylum level to most accurately compare the MAGs to the cultures and amplicons. A full list of taxonomies can be found in Supplementary S2.

Whole genomes of bacterial isolates were also mapped to the seven metagenome assemblies to investigate the shared genetic material between the two methods. Bowtie2 (Langmead and Salzberg 2012) was used to index the whole genomes to the assemblies and reads per million was calculated (Equation 1).

### rRNA gene data handling

Metagenomics reads were processed with an in-house rRNA pipeline (https://github.com/AU-ENVS-Bioinformatics—https://zenodo.org/badge/latestdoi/546561474). Briefly, the raw reads were trimmed, then sorted into a “SSU bin,” assembled into full length rRNA genes and annotated. Full specifications of the programs used, including their versions and options are listed in the Github repository (https://github.com/AU-ENVS-Bioinformatics/TotalRNA-Snakemake).

Analysis of amplicon sequencing data was done using QIIME2-2021.8 (Bolyen et al. 2019), with the Silva 138_99 database (Quast et al. 2013). Samples were not rarefied, as rarefaction curves (Figures S6 and S7, Supporting Information) showed that sufficient sequencing depth was achieved for all samples. Taxonomy data from GTDB was manually checked and corrected to match NCBI taxonomy at phylum level. A full list of taxonomies is available in Supplementary Information S2. Amplicon sequence variants (ASVs) or 16S rRNA genes belonging to chloroplasts or mitochondria were discarded from amplicon and MGS results. Unclassified ASVs were pooled under “other.”

Ampvis2 (Andersen et al. 2018) was used to create heat maps and alpha diversity plots. Shannon Equitability Index was calculated according to Equation (2).


(2) \begin{eqnarray*}
{E}_H = {\mathrm{ }}H/\ln \left( S \right),
\end{eqnarray*}

where H = Shannon diversity index and S = number of ASVs.

Metacoder (Foster et al. 2017) was used for the creation of heat trees, showing diversity at multiple taxonomic levels at the same time. Both packages were used in R Studio 2021.09.01 (R Core Team 2021). Scripts are accessible in the Github repository (https://github.com/AU-ENVS-Bioinformatics/GR21_Greenland_Ice_Sheet_Microbial_Diversity_Data_Handling) (Jaarsma 2023).

All metagenome and bacterial isolate 16S rRNA genes were aligned using MAFFT v7.490 (Katoh et al. 2002) in Geneious Prime on RAxML (Stamatakis 2014). The GTR GAMMA model was used, under the “Rapid Bootstrapping and search for best-scoring ML tree” algorithm, using 100 bootstrap replicates. Bootstrap values lower than 80 were removed.

## Results

### Sequencing output for amplicons and metagenomes

**Figure 3.**
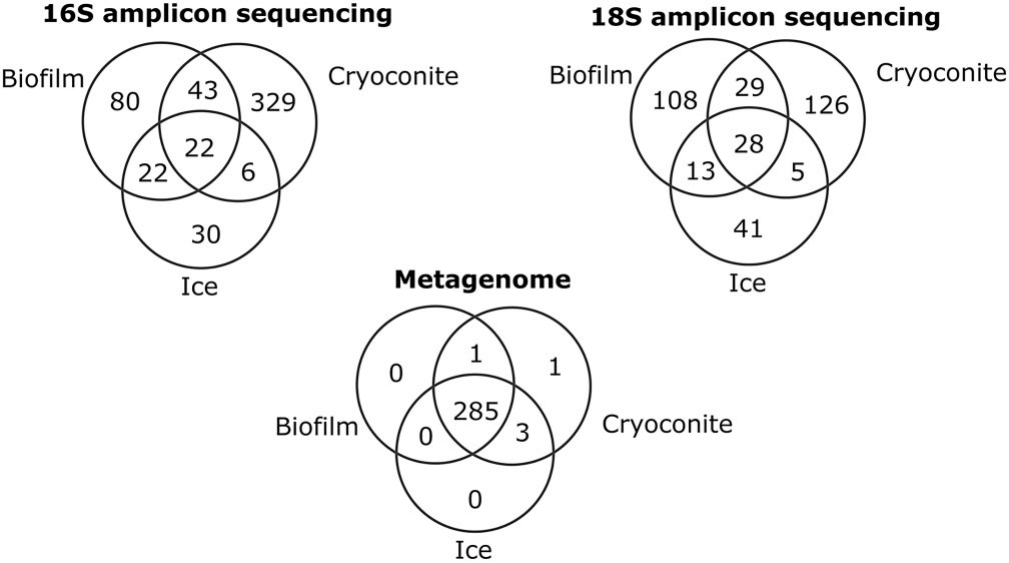
Venn diagrams showing distribution of amplicon sequencing ASVs and metagenome SSU rRNA genes by environment.

After quality checking, most merged reads were produced from the biofilm sample in both 16S and 18S rRNA gene sequencing, followed by cryoconite and ice (Table 1). Most ASVs were, however, obtained from cryoconite, followed by biofilm and ice (Fig. 3). Amplicon sequencing yielded 30 bacterial and 18 eukaryote phyla. Metagenome SSU rRNA gene observations mostly came from ice, followed by cryoconite and biofilm. There was a large overlap in microbial diversity between the three environments using these genes (Fig. 3). The metagenome SSU rRNA genes belonged to 24 bacterial and 12 eukaryote phyla. There were 133 high quality MAGs assembled from the metagenome data. The total number of MAGs varied by sample type. The cryoconite metagenome gave the highest number of MAGs (*n* = 89), while the ice surface and biofilm metagenome assemblies resulted in 32 and 12 MAGs, respectively (Table 1).

**Table 1.** Sequencing output *only one biofilm replicate was used for MGS.

	Ice	Cryoconite	Biofilm	Total
16S amplicon sequencing reads	21 318	56 957	62 974	141 249
18S amplicon sequencing reads	62 785	63 048	80 341	206 174
Metagenome assembled SSU rRNA gene abundance	876 670	256 452	88 480*	1 221 602
Number of MAGs	32	89	12	133

### Alpha diversity in supraglacial habitats

Shannon diversity, Shannon equitability, and inverse Simpson alpha diversity indices, for both amplicon sequencing and the rRNA genes from MGS, were calculated and grouped by sample type (Figure S3, Supporting Information). Based on the Shannon diversity and inverse Simpson indices of rRNA genes from the metagenomes, cryoconite had the highest diversity, followed by biofilm and ice. A similar trend was found for 16S rRNA gene amplicons, but the biofilm and ice were more similar here. 18S rRNA gene amplicon alpha diversity was typically lower compared to those of the 16S rRNA gene. The biofilm sample had the highest eukaryote diversity, followed by cryoconite and ice. Based on the Shannon equitability index, evenness was highest in cryoconite, followed by biofilm and ice.

### 16S and 18S rRNA gene amplicon sequences

**Figure 4.**
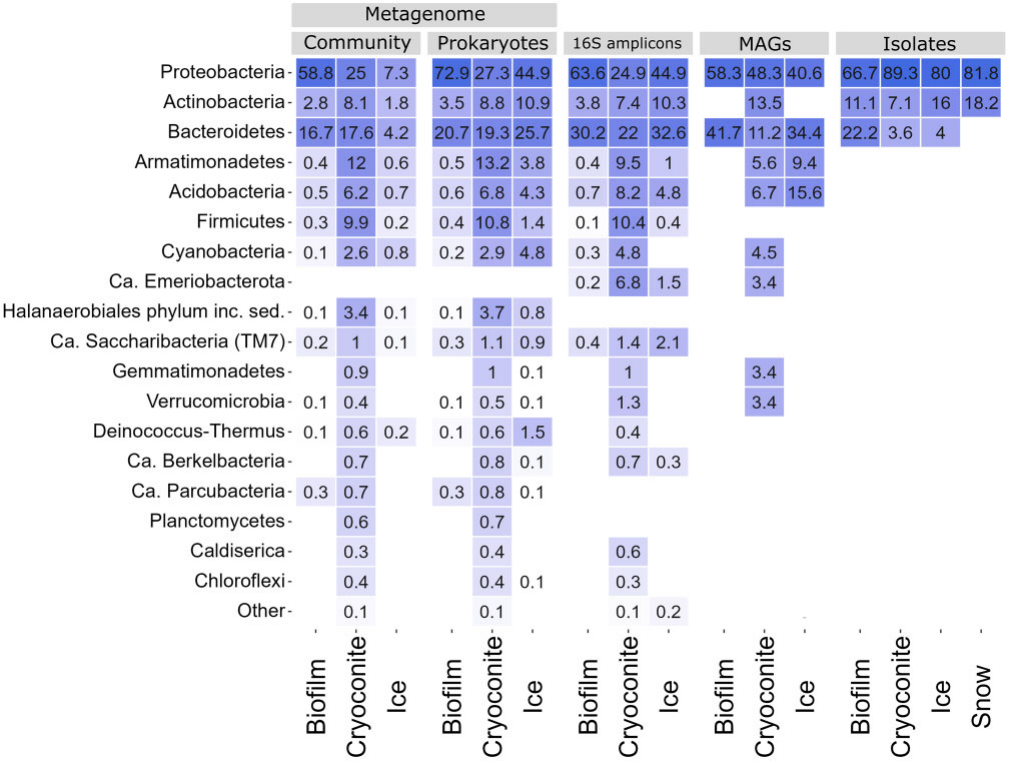
Heat map showing prokaryote relative abundance data from metagenome and amplicon sequencing, abundance of MAGS and cultured isolates, all faceted by the habitat the sample came from. The top 19 most abundant phyla are shown. For the metagenome data, the relative abundance is plotted for both the whole community and prokaryotes only.

The relative abundance of the prokaryotic communities derived from the 16S rRNA amplicon sequencing is plotted as a heatmap showing the top 19 most abundant phyla (Fig. 4). Proteobacteria 16S rRNA genes were the most dominant among bacterial amplicon sequencing results, with a relative abundance of 63.6%, 24.9%, and 44.9% for biofilm, cryoconite, and ice, respectively. Bacteroidetes 16S rRNA genes were also prominent, with a relative abundance of 30.2%, 22.0%, and 32.6% for biofilm, cryoconite, and ice. Actinobacteria 16S rRNA genes amounted to 3.8% of the relative abundance in biofilm, 7.4% in cryoconite, and 10.3% in ice. Firmicutes (10.4%), Armatimonadetes (12.0%), and Acidobacteria (8.2%) 16S rRNA genes were relatively abundant in cryoconite amplicon sequencing data.

**Figure 5.**
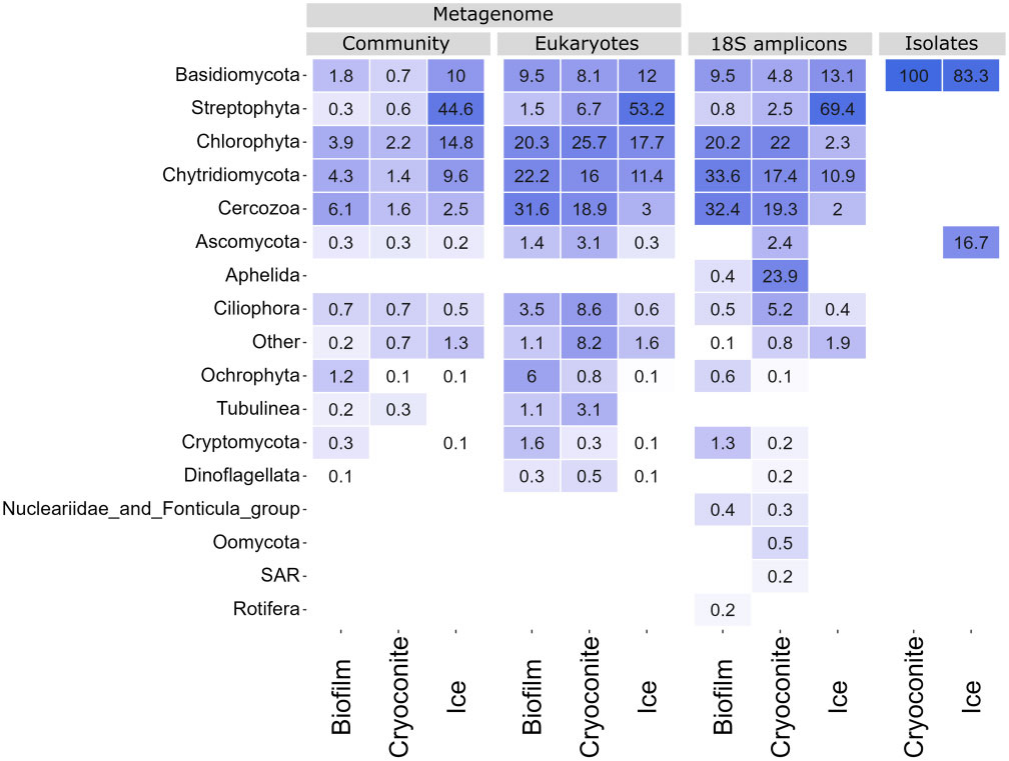
Heat map showing eukaryote relative abundance data from metagenome and amplicon sequencing, and cultured isolates, all faceted by the habitat the sample came from. The top 17 most abundant phyla are shown. For the metagenome data, the relative abundance is plotted for both the whole community and eukaryotes only.

Among the eukaryotic community, Streptophyta 18S rRNA genes were by far the most abundant in ice (69.4%), and less represented in biofilm (0.8%) and cryoconite (2.5%) (Fig. 5). Chytridiomycota 18S rRNA genes had the highest relative abundance in biofilm (33.6%) but were also well-represented in cryoconite (17.4%) and ice (10.9%). Chlorophyte algae 18S rRNA genes had higher relative abundance in biofilm (20.2%) and cryoconite (22.0%) than in ice (2.3%). The same was true for Cercozoa 18S rRNA genes, with 32.4%, 19.3%, and 2.0% for biofilm, cryoconite, and ice, respectively. Aphelida 18S rRNA genes were abundant in cryoconite (23.9%) but much less in the other sample types.

### Metagenome-extracted SSU rRNA genes

**Figure 6.**
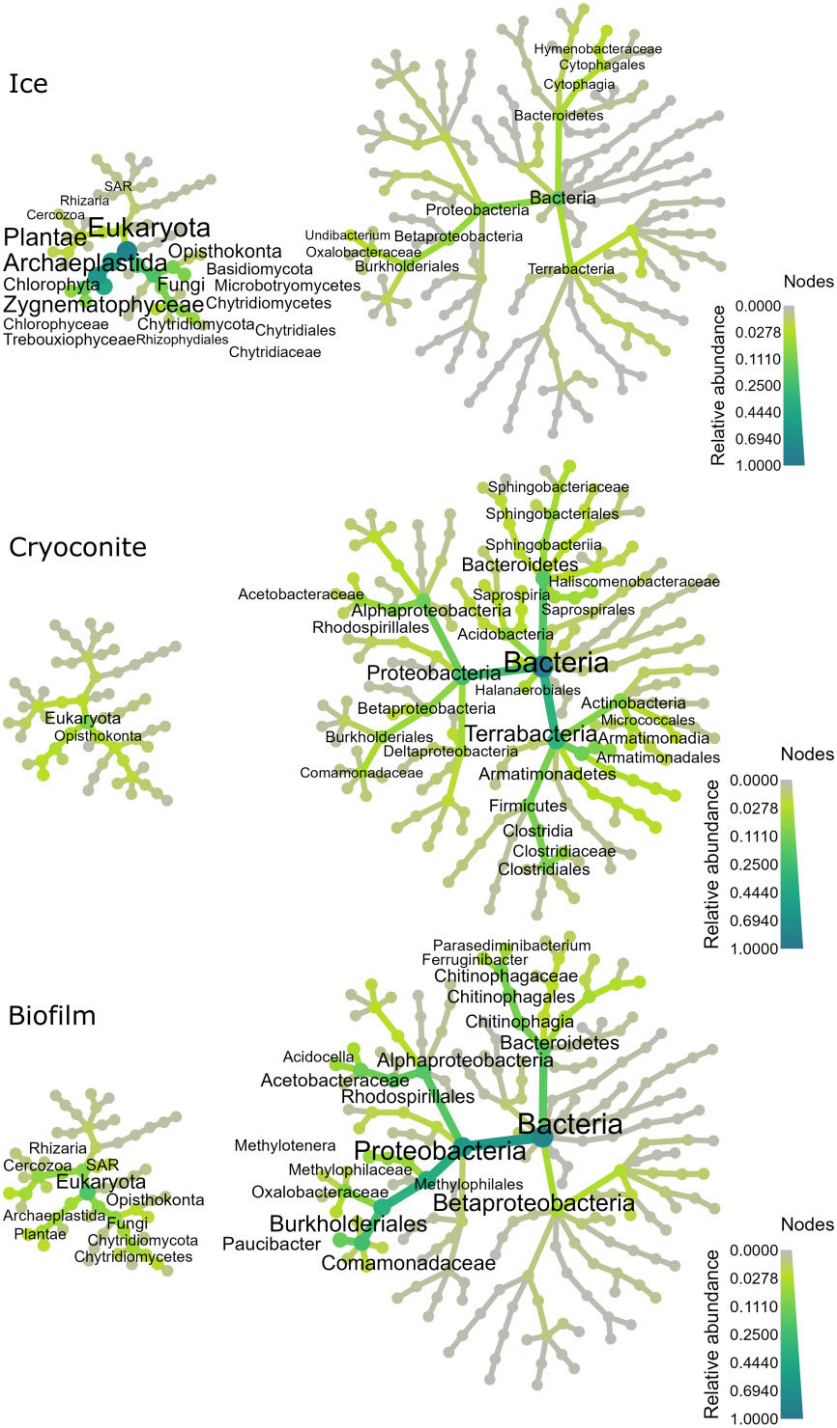
Heat trees plotted using metagenome-extracted rRNA genes. Node size and color correspond to the relative abundance. The top thirty most abundant taxa are labeled, and their text size also correlates with relative abundance.

The abundance of assembled SSU rRNA genes from the metagenomes is plotted as heat trees for each of the three main habitats studied here: ice, cryoconite, and biofilm (Fig. 6). The ice surface eukaryotic microbiome was dominated by Zygnematophyceae, a class of Streptophyte algae, and Chytridiomycota fungi, as determined from 18S rRNA gene sequences. The bacterial community of the ice surface mainly consisted of Proteobacteria, Bacteroidetes, and Terrabacteria, the latter a taxon that encompasses phyla like Armatimonadetes, Firmicutes, and Actinobacteria. The same bacterial taxa dominated the cryoconite and biofilm 16S rRNA genes. The biofilm bacterial community was clearly dominated by Proteobacteria 16S rRNA genes. The biofilm had a relatively higher total of eukaryote rRNA genes compared to bacterial rRNA genes than the cryoconite sample had. Glacier ice algae and Chytridiomycota 18S rRNA genes were again observed among the common eukaryotes in the biofilm sample.

SSU rRNA relative abundances were also plotted as heatmaps (Figs. 4 and 5), grouped by sample type, and revealed the 19 (prokaryotic) and 17 (eukaryotic) most abundant phyla. Among prokaryote and eukaryote SSU rRNA genes combined, Proteobacteria 16S rRNA genes were the most abundant, comprising 58.8%, 25%, and 7.3% of the biofilm, cryoconite, and ice samples, respectively (Fig. 4). The second dominant bacterial phylum was Bacteroidetes, with 16.7%, 17.6%, and 4.2% of SSU rRNA genes for biofilm, cryoconite, and ice, respectively. Actinobacteria amounted to 2.8%, 8.1%, and 1.8% of SSU rRNA genes in biofilm, cryoconite, and ice samples. The evenness of bacterial abundance was higher in cryoconite, where Armatimonadetes (12%), Acidobacteria (6.2%), and Firmicutes (9.9%) also held a substantial share of the SSU rRNA biodiversity. The relative abundance numbers of 16S rRNA genes of these phyla within the prokaryotic communities were comparable to those observed through 16S amplicon sequencing.

For eukaryotic SSU rRNA, the Streptophyta were the most dominant on the ice surface, amounting to 44.6% of total microbial community SSU rRNA genes (Fig. 5). In contrast, these Streptophyta SSU rRNA genes were less dominating in the biofilm (0.3%) and cryoconite (0.6%) samples. In addition, in contrast to the 18S rRNA amplicon sequencing results, Chlorophyta snow algae SSU rRNA genes were also abundant in the dark ice sample (14.8%), yet they were less common in the biofilm (3.9%) and cryoconite (2.2%). Similarly, ice contained 10% Basidiomycota SSU rRNA genes, while the biofilm and cryoconite both contained < 2%. Finally, Chytridiomycota are found in the ice surface sample with 9.6% relative abundance of SSU rRNA genes. Lower abundances were observed for the biofilm (4.3%) and cryoconite (2.2%) samples. A total of four different orders of Chytridiomycota were detected in the metagenome. Ice and biofilm both had a similar profile of chytrids, dominated by *Chytridiales* (67.0% and 71.4% of the total chytrids sequences, respectively), which made up a smaller portion of the cryoconite chytrid community (37.6%). Chytrids found in cryoconite mainly belonged to *Rhizophydiales* (38.4%), which were less abundant in ice (26%) and biofilm (19.1%). *Lobulomycetales* made up 17.9% of the cryoconite chytrids community, but only 3.8% of that of ice, and 3.3% of that of biofilm.

### MAGs

The 133 obtained MAGs were grouped by sample type, and their proportions are shown together with the metagenome and amplicon sequencing results (Fig. 4). The MAGs belonged to nine unique phyla (Table 2). Proteobacteria made up the majority of MAGs (63), followed by Bacteroidetes (26). The majority of MAGs were assembled from the cryoconite metagenome. The 12 biofilm MAGs were made up of only 5.3% of the assembled contigs from the biofilm assembly. The 89 total cryoconite MAGs made up 6.4% (33 MAGS), 4.4% (28 MAGs), and 4.7% (28 MAGs) of the three cryoconite assemblies. The 32 MAGs from the ice surface made up 3.5% (11 MAGs), 2.1% (8 MAGs), and 3.6% (12 MAGs) of the three assemblies from the ice sample.

**Table 2.** Obtained MAGs, grouped by phylum and environment.

Phylum	Cryoconite	Ice	Biofilm	Total
Acidobacteria	6	5		11
Actinobacteria	12			12
Armatimonadetes	5	3		8
Bacteroidetes	10	11	5	26
Ca. Eremiobacterota	3			3
Cyanobacteria	4			4
Gemmatimonadetes	3			3
Proteobacteria	43	13	7	63
Verrucomicrobia	3			3
Total	89	32	12	133

**Figure 7.**
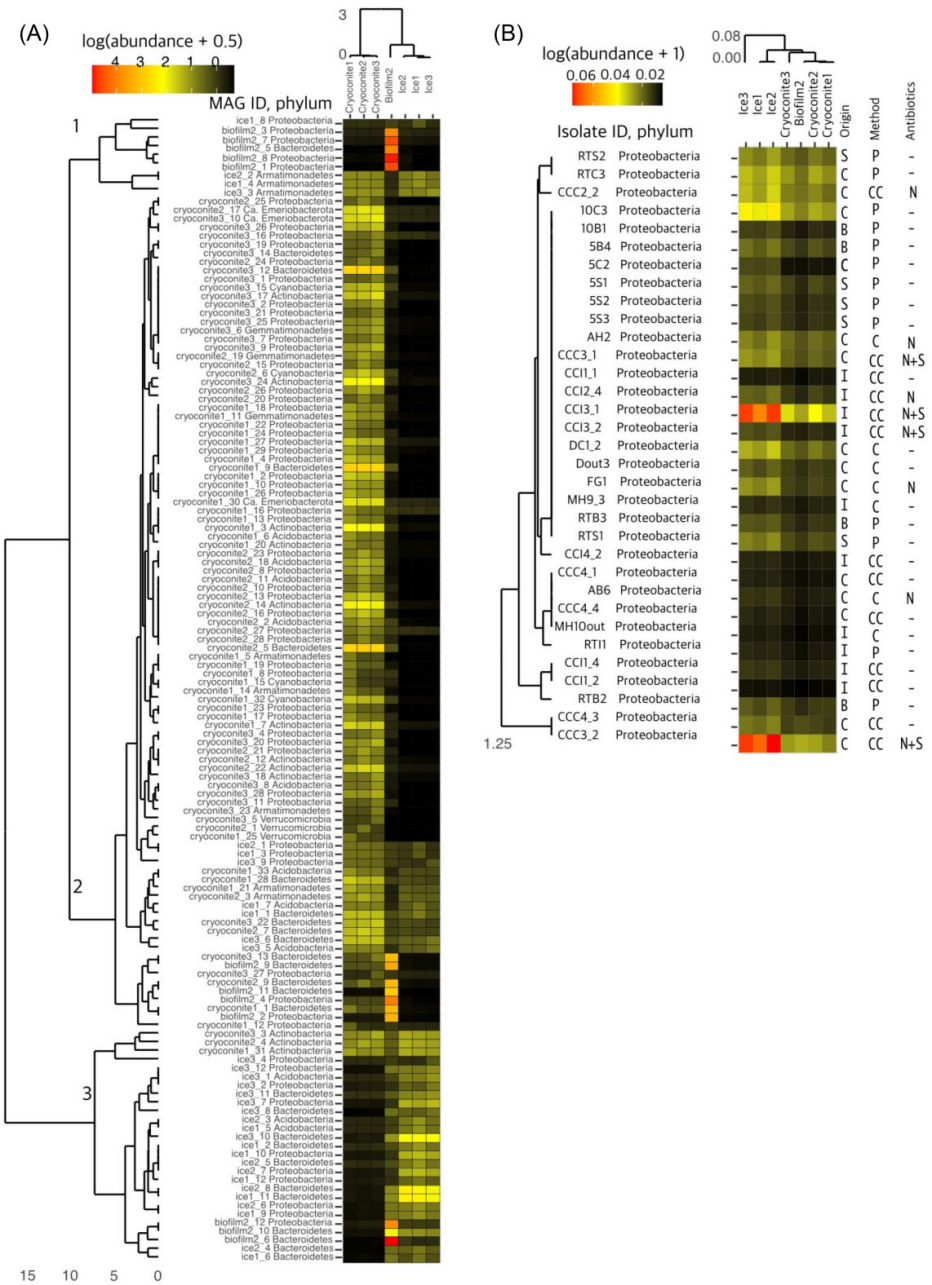
Heatmap of metagenomic contigs mapped between (A) MAGs and (B) isolate genomes in unit of “reads per million” (Equation 1), which normalizes the sum of the mapped contigs by the length of the gene to one million. Hierarchical clustering depicts Spearman rank correlation of the (meta)genomes and the sample sites. Mapped reads are normalized with (A) abundance + 0.5 and (B) abundance +1 to distribute values in a positive scale. Right side of panel (B) includes culture information; Origin: S = snow, C = cryoconite hole, I = ice, and B = biofilm. Method: P = plate (see methods) CC = culture chamber, C = culture chip. Antibiotics:—= none, N = nystatin, N+S = nystatin + streptomycin. Heatmap depiction inspired by Rogers et al. (2023).

Read mapping (see Equation 1) was used to determine if genomic material was shared between MAGs and sample types that they did not originate from. Since the MAGs were single sample assembled, the reads from the individual samples did not mix and the MAGs that originate from one sample type can be confidently assumed to be present in that sample. To further check for patterns in MAG occurrence across the three sample types, the relative abundances of MAGs were clustered by Spearman rank correlation and depicted with a dendrogram. The seven samples were ordered similarly (Fig. 7). Three cocorrelating clusters arose in the MAGs analysis, showing MAGs that were most abundant in the biofilm, cryoconite, or the ice surface assemblies (Fig. 7A). The Spearman correlation clustering showed three groups, widely based on origin sample type. Cluster 1 is biofilm originating MAGs and cluster together because of their high abundance in the biofilm sample. Cluster two contains many MAGs that have abundance in all sample types but also clusters mainly in the cryoconite sample types. Lastly, cluster three is a mix of MAGs that were abundant everywhere and predominant in the ice surface samples.

### Cultured isolates

There were 160 isolates identified through Sanger sequencing. Of the 88 bacterial isolates, 45 were cultured using *in situ* methods (culture chips and chambers), whereas almost all the yeast isolates were obtained using *in situ* methods (Table 3). Only two out of 72 yeast isolates originated from plates. The majority of isolates was obtained from cryoconite, followed by ice, snow, and biofilm.

**Table 3.** Distribution of 160 identified isolates by culture method and environment.

	Bacteria				Yeasts	
Culture chip	19	7	N/a	N/a	65	2
Culture chamber	11	8	N/a	N/a	1	2
Plate	10	12	10	11	0	2
	**Cryoconite**	**Ice**	**Biofilm**	**Snow**	**Cryoconite**	**Ice**

Whole genomes of 73 bacterial isolates were sequenced, after which full length 16S rRNA genes were extracted. BLASTn was used to identify the closest relative in the NCBI database. A table of all isolate information, including closest relatives (highest % identity determined through Sanger sequencing and whole-genome sequencing), can be found in Supplementary S4. The culturing method and sample origin of each isolate are also listed in this table. Only 33 of the isolated whole genomes showed nonzero read abundance in the metagenome libraries, albeit low in general (Fig. 7). The only isolates to have reads mapped to the metagenome sample types belonged to the phylum Proteobacteria. Only two of the isolates that originated from the culture chamber method (from ice and cryoconite; Fig. 7B) had a high read mapping to the ice surface samples, which was nearly six times higher than the reads mapped from the other cultures. Additionally, the isolates did not show any clustering pattern based on sample origin in the same way the MAGs did, but instead showed a general presence or absence within the seven metagenome libraries.

The full length 16S rRNA genes were also used to plot abundance of bacterial isolates in a heatmap (Fig. 4), whereas LSU Sanger sequencing results were used to plot the abundance of yeast isolates (Fig. 5). The 73 bacterial isolates from which a full genome was obtained belonged to 10 genera within Proteobacteria, Bacteroidetes, and Actinobacteria. Proteobacteria made up the majority (*n* = 60) of isolates, with 43 isolates belonging to *Pseudomonas. Herbaspirillum* and *Cryobacterium* were two additional abundant genera, with nine and seven isolates, respectively. In total, 28 of the 73 bacterial isolates were cultured from the cryoconite sample, 25 from ice, 11 from snow, and nine from the biofilm. Yeasts were only obtained from the ice and cryoconite samples. A total of five yeast genera were observed, belonging to two phyla, Basidiomycota and Ascomycota. *Mrakia* (*n* = 46) and *Camptobasidium* (*n* = 23) made up the bulk of the isolates (Fig. 5). A phylogenetic tree of isolate and metagenome 16S rRNA genes is available in supplementary information S5.

## Discussion

In this study, we mapped the microbial diversity of supraglacial habitats on the Greenland Ice Sheet. Using different sequencing methods, we identified different dominant community members. Subsequently, the combination of (metagenome) sequencing and the culturing approach enabled comparison of obtained genomes (MAGs or isolate genomes) to these dominant community members. Using read mapping, we visualized the genetic overlap between the obtained genomes and the metagenomes.

### The Greenland Ice Sheet contains a variety of life

The Greenland Ice Sheet contains abundant microbial life in all its habitats (Anesio et al. 2017). With the culture-dependent and culture-independent work presented here, we expand this catalog with organisms from four environmental sample types: cryoconite, biofilm, surface ice, and snow. We documented and confirmed several keystone species within these respective habitats. For example, an important ecosystem engineer in cryoconite holes, the cyanobacterium *Phormidesmis priestleyi* (Gokul et al. 2019), constituted ∼5% of the MAGs acquired from cryoconite. The ice surface sample was dominated by eukaryote SSU rRNA genes, mainly from the Streptophyte algae Zygnematophyceae, which composed 45% and 69% of the metagenome and amplicon sequencing rRNA genes, respectively. This is not surprising, considering that these dark pigmented glacier ice algae can form large blooms on the ice surface over the summer (Williamson et al. 2020). While our data does not reflect abundance of live cells directly, the previously described relatively high counts of algal cells on the Greenland Ice Sheet (Holland et al. 2019) corroborates our high rRNA gene abundance data. Furthermore, it has been found that, on average, 13.5% of cells in supraglacial meltwaters are larger than 10 µm, and therefore likely belong to either cyanobacteria or eukaryotic glacier ice algae (Stevens et al. 2022).

Although the role of glacier ice algae in the darkening and melt of the ice has been demonstrated (Chevrollier et al. 2022), their interactions with other members of the ice microbial community have rarely been described. Chytridiomycota have been suggested to be associated with algal blooms as parasites, perhaps providing a top-down control on the glacial ice algae (Perini et al. 2022). The same interaction was documented in Alaskan cryoconite holes, and it was suggested that cryoconite holes could be hotspots for chytrid infections (Kobayashi et al. 2023). In the present study, we found chytrids to be abundant in the eukaryote rRNA gene data in both biofilm and cryoconite, but surprisingly to a lesser extent in the ice surface sample. The chytrid communities also differed in the sample types, raising the question if chytrids in cryoconite holes parasitize different targets than those on the ice surface or in biofilms.

Furthermore, predatory protists like Cercozoa and Ciliophora were observed in all sample types. The Cercozoa family Vampyrellidae was 1.8 times more abundant in biofilm than cryoconite, and 2.7 times more abundant in biofilm than ice. This algivore is known to perforate and feed on Zygnematophyceae and Chlorophyceae cells (Hess et al. 2012). Vampyrellidae have been observed in polar cryoconite before (Millar et al. 2021). It would be worthwhile to investigate further whether this protist is one of the biological controls of the glacier ice algae together with chytrids. The biofilm was also rich in rRNA genes from Chrysophyceae, golden algae, which were much less abundant in cryoconite (17.9 times less) and ice (3.9 times less). The biofilm observed in this study might thus be formed by detritus from ice surface eukaryotes that get washed into the cryoconite holes. This is also supported by the brown–red color of the biofilm, which likely originates from algal pigment. Glacier ice algae SSU rRNA genes were scarce in the biofilm. Further study is needed to confirm if they are consumed by chytrids or protists in the biofilm, which could explain the lack of observed genes due to their fast utilization as a food source.

The dominant bacterial phyla observed in this study match similar amplicon sequencing studies. Proteobacteria, Bacteroidetes, Actinobacteria, and Cyanobacteria are frequently reported as abundant in supraglacial habitats of the Greenland Ice Sheet (Musilova et al. 2015, Stibal et al. 2015, Perini et al. 2019, Millar et al. 2021). Cryoconite samples harbor the highest microbial diversity among the other supraglacial habitats sampled in this study. This is in correspondence with previous studies that compare different supraglacial habitats, for instance on Tibetan glaciers (Liu et al. 2022). No significant differences in alpha diversity of ice and cryoconite were however observed by others (Cameron et al. 2016).

### Metagenome approach complements amplicon sequencing to investigate microbial biodiversity

In the current study, we investigated the microbial diversity of supraglacial habitats on the Greenland ice sheet using various culturing-dependent and -independent approaches. This allows for comparison of the effectiveness of these different methods for capturing this diversity. Using rRNA genes from MGS offers a few advantages over using amplicon data because of procedural differences. The choice of primer pair can influence the reflection of the diversity within short read amplicons (Lewis et al. 2020). Using the metagenome approach, the introduction of primer bias in the data is, however, avoided because the amplification step targets the adapter ligated DNA fragments, thus avoiding preferential binding. Another benefit is the fact that full length SSU rRNA genes are obtained through MGS and assembly. A higher resolution for phylogeny assignment is thus achieved compared to amplicon sequencing, which relies on certain regions of the rRNA genes, unless long read amplicons are used (Johnson et al. 2019).

A lower diversity of SSU rRNA gene taxa was observed in the metagenome than through amplicon sequencing, but they were more evenly distributed among the sample types in the metagenome. Despite a higher sequencing depth, the SSU rRNA genes make up only a small portion of the total metagenome (0.27%) and are thus relatively less covered. Low abundant organisms may, therefore, be overlooked. It is difficult to assess to which extent this has happened, and if sequencing depth should have been even greater. Total RNA sequencing can be considered as an alternative that requires lower sequencing depth, while still allowing representation of the whole community (Cottier et al. 2018). It has been previously discussed that intragenomic copies of 16S rRNA genes can lead to artificially high diversity when an ASV approach is taken, falsely splitting them up into different taxa. Conversely, closely related but slightly different SSU rRNA genes may have been assembled together in the metagenome approach, leading to an underestimation of diversity (Johnson et al. 2019).

A classic amplicon approach yields two different datasets, as eukaryote and prokaryote rRNA genes are amplified in separate PCR reactions. Rather than using two separate bar plots to visualize the relative abundances of the 16S and 18S rRNA genes, which is a common way to present amplicon data, we demonstrate here the use of heat trees (Fig. 6) to visualize differences in prokaryote/eukaryote gene proportions in different environments. This illustrates a powerful benefit of MGS over amplicon sequencing, i.e. the direct comparison of rRNA gene relative abundances of the entire community. This provides valuable information for the study of ecological relations between members of this community, although it should be kept in mind that the abundance of rRNA genes is a mere proxy for the abundance of active cells in the environment. The ice surface sample contained more eukaryote than prokaryote SSU rRNA genes. Although copy numbers of rRNA genes are generally higher for eukaryotes (Hori et al. 2023), this could indicate a higher number of eukaryotes on the ice surface. Despite generally higher copy numbers in eukaryotes, the biofilm and cryoconite samples contained more prokaryote SSU rRNA genes. Based on this approach, we demonstrate the relatively higher importance of the eukaryotic community compared to the prokaryotic community on the ice surface, while the opposite is true in cryoconite holes and the biofilm.

### Distribution of MAGs

Assembling MAGs can reveal microbial dark matter in the environment. It is, however, clear that most of the metagenomic assembly is unused; only a small portion of contigs from the metagenome contributed to the assembly of high-quality MAGs. This can be partially explained by the fact that only high quality bacterial MAGs were assembled in this study, ignoring the eukaryotic DNA in the assembly. The MAGs that originated from a single sample type typically had some presence in the other two sample types. Spearman correlation of MAG read mapping abundances showed that some of the MAGs were unique to their origin source, while others have some presence in the other sample types. This emphasized that there are some organisms that are isolated to a unique environment through environmental filtering, whereas others may be more generic ice sheet microbes. In the heatmap (Fig. 7), there is a group of MAGs that maps back to both cryoconite and ice, regardless of which metagenome they were assembled from. This group includes the genera *Granulicella, JAFAZD01, Lacisediminihabitans*, the families *Sphingomonadaceae, Capsulimonadaceae*, and the order *Sphingobacteriales*. Examples of more specialist taxa include *P. priestleyi*, which was only acquired as a MAG from cryoconite. A collection of MAGs appears to be predominantly associated with biofilm. This group includes the (genomo)genera *Rhodoferax, Sediminibacterium, Methylotenera, Palsa-911, Paucibacter, Arcicella, CAIQQQQ01*, and *JAAFHG01*.

Several MAGs that could be classified to genomospecies level have been observed before. Three *Ferruginibacter sp014377975* MAGs, assembled from the cryoconite metagenome but abundant in biofilm, have previously been obtained as MAG from a Greenlandic glacier (Genbank accession number GCA_014377975.1). Genome assemblies of *Gemmatimonadaceae sp. AG11 sp014378185* (Genbank accession number GCA_014378185.1), and *Undibacterium sp014376575* (Genbank accession number GCA_014376575.1) were reported in the same study (Bioproject accession number PRJNA552582). *Sphingomonas psychrolutea*, abundant in biofilm, has previously been found in Tibetan glacier ice samples (Liu et al. 2015).

On Tibetan glaciers, the majority of genomes (either from isolates or MAGs) were obtained from cryoconite, followed by ice and snow (Liu et al. 2022). Mostly Proteobacteria were observed, followed by Actinobacteria, Bacteroidetes, and Firmicutes. In contrast to our findings, it was observed that the majority of what was cultivated could also be obtained as MAGs (Liu et al. 2022). Isolates in that study were incubated at 4°C in R2A broth. Another metagenome study of Greenland Ice Sheet cryoconite obtained 29 MAGs, 13 of which were 100% complete, and included a Cyanobacterium, Proteobacteria, Actinobacteria, Bacteroidetes, Acidobacteria, and Chloroflexi (Hauptmann et al. 2017).

### Recovery of isolate genomes

Culturing approaches are known to only capture a small representation of the microbes present in the natural environment, which leaves most of the organisms unknown (Lloyd et al. 2018). A recent study found that, of the microbial diversity of Svalbard permafrost observed through amplicon sequencing, only 6.37% of bacterial and 20% of fungal genera observed through amplicon sequencing were cultivable (Dziurzynski et al. 2022). It is estimated that less than 1% of the total environmental microbial diversity can be cultivated under laboratory conditions (Kaeberlein et al. 2002). When isolates are the only source of information, most diversity is thus missing, as also demonstrated in this study. There is less diversity in isolates than there is in MAGs. This is expected, as MGS circumvents cultivation bias. The observed MAGs might still be biased toward more abundant taxa, despite high sequencing depth. The biofilm MAGs *Ferruginibacter, Methylotenera*, and *Paucibacter* for instance also appear abundant in the 16S rRNA extracted genes (Fig. 6). Only four genera overlap between MAGs and isolates: *Sphingomonas, Rhodoferax, Rhodanobacter*, and *Lacisediminihabitans*.

Many cultured isolate taxa in this study match those previously reported. Proteobacteria, Actinobacteria, and Bacteroidetes, which encompass the isolates in this study are commonly found, albeit in varying ratios. In a review of 340 bacterial strains isolated from glacial habitats around the globe, Proteobacteria are the most dominant, followed by Actinobacteria, Firmicutes, Bacteroidetes, and Deinococcus-Thermus (Kim et al. 2022). The yeast genera *Mrakia, Dothiora, Phenoliferia*, and *Camptobasidium* have been previously isolated from Greenland (Perini et al. 2019, 2021).

Read mapping the abundance of the isolate genomes to the seven metagenome assemblies shows that the culturable organisms represented a small proportion of the total environmental DNA (Fig. 7B). Only 33 of the 73 isolate whole genomes had a nonzero amount of contigs map to the assembled metagenome reads. The whole genomes that had presence in the metagenome assemblies were all from the phylum Proteobacteria, which was also the most abundant phylum in the MAGs. This dominance of Proteobacteria could be because this phylum is the dominating microbe in many cryospheric environments (Bourquin et al. 2022), or because current methods in DNA identification are biased toward cultured representatives (Lloyd et al. 2018). When there are no mapped reads shared between the metagenome assembly and the (meta)genome’s contigs, we can assume that there is little to no presence of that genome in the sample assembly. The isolates, while belonging mostly to the generally abundant Proteobacteria, seem rare in the supraglacial environment. In the phylogenetic tree of isolate and metagenome 16S rRNA genes (Figure S5, Supporting Information), the large group of *Pseudomonas* isolates clustered together with only one *Pseudomonas* 16S rRNA gene from the metagenome. The amplicon data only yielded two *Pseudomonas* ASVs, both only found in ice. Similarly, the *Mucilaginibacter* isolates did not cluster together with metagenome 16S rRNA genes. *Mucilaginibacter* was, however, observed in the amplicon sequencing results, with four different ASVs. While relatives of the other isolates were observed in the phylogenetic tree, most isolates are apparently not representing the most abundant genera in the environment.

Judging from the read mapping, the sample origin of the isolates does not always match the actual abundance in the metagenome from that habitat, whereas this is more predictable for the MAGs. There is thus a degree of randomness in observed isolates from the different samples. As a result, the obtained cultured isolates do not necessarily reflect the most abundant organisms in the habitat they were sampled from. Conflicting studies exist that cover the presence of distinct communities in habitats such as ice and cryoconite. In one study, spatial distribution across the ice surface seemed to be the main component of variability in bacterial communities, while differences between ice and cryoconite communities seemed less distinct (Cameron et al. 2016). Others have, however, demonstrated that there can be significant differences in community between the ice surface and cryoconite hole habitats (Musilova et al. 2015). Simultaneously, it is shown that many taxa are specific to a certain environment (Perini et al. 2019), which is also observed in the read mapping of the MAGs against the metagenomes in this study.

The habitats sampled in this study are in close spatial proximity to each other and connected through flow of liquid water in the melt season (Cameron et al. 2020). It has recently been suggested that microbes—and especially bacteria—can be transported through the weathering crust, for instance from and into cryoconite holes (Cook et al. 2016), in particular considering that surface meltwaters consistently contain about 10^4^ cells ml^−1^ (Stevens et al. 2022). The controls on transport of microbial cells through the weathering crusts remains, however, one of the outstanding questions (Halbach et al. 2023). The standing theory for inoculation of the supraglacial environments by bacteria is that they are delivered through aerial deposition (Šantl-Temkiv et al. 2018, Cameron et al. 2020). Once on the ice sheet, a significant portion of glacier surface microbes are subsequently found to be metabolically inactive (Bradley et al. 2022). This means certain taxa of microbes observed in this study through culturing might spring from transient cells in the sample they were captured from, rather than settled and thriving colonies.

Detection of these cells through sequencing would prove difficult, since there is a minimum amount of biomass needed to detect their DNA. However, during culturing, even low abundant bacterial cells are given a chance to grow into a colony, after they are provided a more stable medium to reside in. This change in conditions may increase the fitness of some isolates that were otherwise less adapted to survive the harsh ice sheet conditions. Particularly in the *in situ* culturing methods used in this study, these cells are given more time, enabling stochastic awakening from dormancy following the “scout model” (Epstein 2009), but also potentially a more favorable set of environmental conditions that could induce awakening from dormancy according to the “comfort timing” strategy hypothesis (Laugier 2023). For the capture of diversity in genomes, it seems therefore beneficial to use a (*in situ)* culturing approach to access transient, dormant, or low abundant microbes if combined with metagenomics studies.

### Evaluation of (*in situ*) culturing

The R2A plates inoculated in the field only yielded eight colonies, a very low amount compared to the 52 obtained through plating that was done under laboratory conditions once samples were returned from the field. The difference between the two approaches was the presence of glycerol in the plates inoculated in the field, to match the medium that was used in the chips and chambers. This study used glycerol as this was essential as an antifreeze agent to protect the integrity of the agar under the field conditions. Therefore, we cannot exclude that if the glycerol had a negative impact on growth of microbial isolates on the plates, it could also have done the same for the culture chips and chambers, since all chips and chambers contained the same concentration of glycerol. It is important to consider that choice of growth medium, as well as incubation conditions and sampling strategy, can influence the resulting cultivable organisms.

The culture chips and chambers kept their integrity while incubating on the ice sheet, despite weather conditions ranging from intense sun to heavy rain, hard wind, and freezing temperatures. Fungal overgrowth and freeze–thaw cycles were foreseen as two challenges during the fieldwork planning phase. Fungal overgrowth was not observed, meaning that the use of nystatin was unnecessary. To protect the structural integrity of the agar gel during repeated freezing and thawing, glycerol was added as an antifreeze agent. This proved to be effective, as the agar in the wells and chambers kept its structure over the entire period. Absence of liquid water, which can be an issue when incubating ichips in soils, was not a problem for the supraglacial environments used in this study. The chips and chambers incubating submerged in the cryoconite hole were more similar to aquatic environments where culture chips have been applied before (Jung et al. 2021). On the ice surface, the chips and chambers were kept above liquid water upon the weathering-crust but received plenty of exposure to hydration from rain and melt water. These natural processes were likely the conduits of nutrient transported through the membranes into the chips and chambers. Applying the chips and chambers on the ice sheet demonstrated that the range of use of *in situ* culturing can be extended further in the cryosphere.

## Conclusion

A variety of complimentary methods (sequencing- and culturing-based) were used to investigate the microbial diversity of supraglacial habitats on the southern area of the Greenland ice sheet. Classic amplicon sequencing approaches were complemented with metagenome data. A clear advantage of the metagenomic approach over amplicon sequencing was the ability to directly compare prokaryote and eukaryote SSU rRNA gene abundances, highlighting in particular the abundance of rRNA genes from eukaryotes over those from prokaryotes on the ice surface, and the opposite in cryoconite holes and biofilm. Two novel *in situ* culturing approaches (culture chips and chambers) have been successfully applied on the surface of the Greenland Ice Sheet. To our knowledge, this is the first time that these techniques have been applied to the challenging glacial environments, such as the ice surface and cryoconite holes. This proof-of-concept paves the way for wider use of *in situ* culturing approaches in the cryosphere.

We demonstrate that different genomic and culturing approaches complement each other. If the objective is to capture as much genetic diversity as possible, for instance in the search for biotechnologically relevant microbes, MAGs complement the genomes obtained through culturing. The assembly of MAGs is biased by abundance in the metagenome, but the isolates obtained do not necessarily reflect abundant members of the community. The genomes obtained as MAG or through whole-genome sequencing thus originate from respectively more, and less established microbes.

These findings highlight the potential benefits of using multiple methods to study microbial diversity in complex environments, particularly for capturing genomic diversity.

## Supplementary Material

fiad119_Supplemental_Files

## Data Availability

Datasets used in this study are available in the NCBI database under BioProject accession number PRJNA942590.

## References

[bib1] Albers CN , Ellegaard-Jensen L, Hansen LH et al. Bioaugmentation of rapid sand filters by microbiome priming with a nitrifying consortium will optimize production of drinking water from groundwater. Water Res. 2018;129:1–10. 29127829 10.1016/j.watres.2017.11.009

[bib2] Andersen KS , Kirkegaard RH, Karst SM et al. ampvis2: an R package to analyse and visualise 16S rRNA amplicon data. Biorxiv. 2018. 10.1101/299537.

[bib3] Anesio AM , Laybourn-Parry J. Glaciers and ice sheets as a biome. Trends Ecol Evol. 2012;27:219–25. 22000675 10.1016/j.tree.2011.09.012

[bib4] Anesio AM , Lutz S, Chrismas NAM et al. The microbiome of glaciers and ice sheets. Npj Biofilms Microbiomes. 2017;3:0–1. 10.1038/s41522-017-0019-0 PMC5460203 28649411

[bib5] Berdy B , Spoering AL, Ling LL et al. In situ cultivation of previously uncultivable microorganisms using the ichip. Nat Protoc. 2017;12:2232–42. 29532802 10.1038/nprot.2017.074

[bib6] Bolyen E , Rideout JR, Dillon MR et al. Reproducible, interactive, scalable and extensible microbiome data science using QIIME 2. Nat Biotechnol. 2019;37:852–7. 31341288 10.1038/s41587-019-0209-9 PMC7015180

[bib7] Bourquin M , Busi SB, Fodelianakis S et al. The microbiome of cryospheric ecosystems. Nat Commun. 2022;13:1–9. 35655063 10.1038/s41467-022-30816-4 PMC9163120

[bib8] Bradley JA , Trivedi CB, Winkel M et al. Active and dormant microorganisms on glacier surfaces. Geobiology. 2022;21:1–18. 10.1111/gbi.12535 PMC10099831 36450703

[bib9] Cameron KA , Müller O, Stibal M et al. Glacial microbiota are hydrologically connected and temporally variable. Environ Microbiol. 2020;22:3172–87. 32383292 10.1111/1462-2920.15059

[bib10] Cameron KA , Stibal M, Zarsky JD et al. Supraglacial bacterial community structures vary across the Greenland ice sheet. FEMS Microbiol Ecol. 2016;92:1–11. 10.1093/femsec/fiv164 26691594

[bib11] Campuzano C. AU-ENVS-Bioinformatics/IlluminaSnakemake. v1.1.0. GitHub. 2023. 10.5281/ZENODO.7648481.

[bib12] Cheung MK , Au CH, Chu KH et al. Composition and genetic diversity of picoeukaryotes in subtropical coastal waters as revealed by 454 pyrosequencing. ISME J. 2010;4 :1053–9. 10.1038/ismej.2010.26 20336159

[bib13] Chevrollier L-A , Cook JM, Halbach L et al. Light absorption and albedo reduction by pigmented microalgae on snow and ice. J Glaciol. 2022;69:1–9.

[bib14] Cook J , Edwards A, Takeuchi N et al. Cryoconite: the dark biological secret of the cryosphere. Prog Phys Geogr. 2016;40:66–111.

[bib15] Cook JM , Hodson AJ, Irvine-Fynn TDL. Supraglacial weathering crust dynamics inferred from cryoconite hole hydrology. Hydrol Process. 2016;30:433–46.

[bib16] Cook JM , Tedstone AJ, Williamson C et al. Glacier algae accelerate melt rates on the south-western Greenland Ice Sheet. Cryosphere. 2020;14:309–30.

[bib17] Cottier F , Srinivasan KG, Yurieva M et al. Advantages of meta-total RNA sequencing (MeTRS) over shotgun metagenomics and amplicon-based sequencing in the profiling of complex microbial communities. Npj Biofilms Microbiomes. 2018;4. 10.1038/s41522-017-0046-x. PMC5773663 29367879

[bib18] Dziurzynski M , Gorecki A, Pawlowska J et al. Revealing the diversity of bacteria and fungi in the active layer of permafrost at Spitsbergen Island (Arctic) – combining classical microbiology and metabarcoding for ecological and bioprospecting exploration. Sci Total Environ. 2022;856:159072. 36179845 10.1016/j.scitotenv.2022.159072

[bib19] Edwards A , Cameron KA, Cook JM et al. Microbial genomics amidst the Arctic crisis. Microb Genomics. 2020;6. 10.1099/mgen.0.000375. PMC7371112 32392124

[bib20] Epstein SS. Microbial awakenings. Nature. 2009;457:1083. 19242455 10.1038/4571083a

[bib21] Fausto RS , Van As D, Mankoff KD et al. Programme for monitoring of the Greenland Ice Sheet (PROMICE) automatic weather station data. Earth Syst Sci Data. 2021;13:3819–45.

[bib22] Feld L , Nielsen TK, Hansen LH et al. Establishment of bacterial herbicide degraders in a rapid sand filter for bioremediation of phenoxypropionate-polluted groundwater. Appl Environ Microbiol. 2016;82:878–87. 26590282 10.1128/AEM.02600-15 PMC4725289

[bib23] Foster ZSL , Sharpton TJ, Grünwald NJ. Metacoder: an R package for visualization and manipulation of community taxonomic diversity data. PLoS Comput Biol. 2017;13. 10.1371/journal.pcbi.1005404. PMC5340466 28222096

[bib24] Gokul JK , Cameron KA, Irvine-Fynn TDL et al. Illuminating the dynamic rare biosphere of the Greenland Ice Sheet's Dark Zone. FEMS Microbiol Ecol. 2019;95:177. 10.1093/femsec/fiz177 31697309

[bib25] Goordial J , Altshuler I, Hindson K et al. In situ field sequencing and life detection in remote (79°26’N) Canadian high Arctic permafrost ice wedge microbial communities. Front Microbiol. 2017;8:1–14. 29326684 10.3389/fmicb.2017.02594 PMC5742409

[bib26] Halbach L , Chevrollier L-A, Cook JM et al. Dark ice in a warming world: advances and challenges in the study of Greenland Ice Sheet's biological darkening. Ann Glaciol. 2023:1–6. 10.1017/aog.2023.17.

[bib27] Hansen CHF , Krych L, Nielsen DS et al. Early life treatment with vancomycin propagates *Akkermansia muciniphila* and reduces diabetes incidence in the NOD mouse. Diabetologia. 2012;55:2285–94. 22572803 10.1007/s00125-012-2564-7

[bib28] Hauptmann AL , Sicheritz-Pontén T, Cameron KA et al. Contamination of the Arctic reflected in microbial metagenomes from the Greenland Ice Sheet. Environ Res Lett. 2017;12. 10.1088/1748-9326/aa7445.

[bib29] Hess S , Sausen N, Melkonian M. Shedding light on vampires: the phylogeny of Vampyrellid amoebae revisited. PLoS ONE. 2012;7:e31165. 22355342 10.1371/journal.pone.0031165 PMC3280292

[bib30] Holland AT , Williamson CJ, Sgouridis F et al. Dissolved organic nutrients dominate melting surface ice of the Dark Zone (Greenland Ice Sheet). Biogeosciences. 2019;16:3283–96.

[bib31] Hori Y , Engel C, Kobayashi T. Regulation of ribosomal RNA gene copy number, transcription and nucleolus organization in eukaryotes. Nat Rev Mol Cell Biol. 2023;2023:1–16. 10.1038/s41580-022-00573-9 36732602

[bib32] Jaarsma A. AU-ENVS-Bioinformatics/GR21_Greenland_Ice_Sheet_Microbial_Diversity_Data_Handling: 1.0.0. GitHub. 2023. 10.5281/ZENODO.7859165.

[bib33] Johnson JS , Spakowicz DJ, Hong BY et al. Evaluation of 16S rRNA gene sequencing for species and strain-level microbiome analysis. Nat Commun. 2019;10:1–11. 31695033 10.1038/s41467-019-13036-1 PMC6834636

[bib34] Jung D , Aoi Y, Epstein S. In situ cultivation allows for recovery of bacterial types competitive in their natural environment. Microbes Environ. 2016;31:456–9. 27682804 10.1264/jsme2.ME16079 PMC5158119

[bib35] Jung D , Liu L, He S. Application of in situ cultivation in marine microbial resource mining. Mar Life Sci Technol. 2021;3:148–61. 37073342 10.1007/s42995-020-00063-x PMC10077220

[bib36] Kaeberlein T , Lewis K, Epstein SS. Isolating “uncultivable” microorganisms in pure culture in a simulated natural environment. Science. 2002;296:1127–9. 12004133 10.1126/science.1070633

[bib37] Kang DD , Li F, Kirton E et al. MetaBAT 2: an adaptive binning algorithm for robust and efficient genome reconstruction from metagenome assemblies. PeerJ. 2019;2019. 10.7717/peerj.7359. PMC6662567 31388474

[bib38] Katoh K , Misawa K, Kuma KI et al. MAFFT: a novel method for rapid multiple sequence alignment based on fast fourier transform. Nucleic Acids Res. 2002;30:3059–66. 12136088 10.1093/nar/gkf436 PMC135756

[bib39] Kim S , Lee H, Hur SD et al. Glaciers as microbial habitats: current knowledge and implication. J Microbiol. 2022;60:767–79. 35904688 10.1007/s12275-022-2275-9

[bib40] Kobayashi K , Takeuchi N, Kagami M. High prevalence of parasitic chytrids infection of glacier algae in cryoconite holes in Alaska. Sci Rep. 2023;13:1–9. 36894609 10.1038/s41598-023-30721-w PMC9998860

[bib41] Langmead B , Salzberg SL. Fast gapped-read alignment with Bowtie 2. Nat Methods. 2012;9:357–9. 22388286 10.1038/nmeth.1923 PMC3322381

[bib42] Laugier J. The “comfort timing” strategy: a potential pathway for the cultivation of uncultured microorganisms and a possible adaptation for environmental colonisation. FEMS Microbiol Ecol. 2023;99:1–7. 10.1093/femsec/fiad026 36921985

[bib43] Lewis WH , Tahon G, Geesink P et al. Innovations to culturing the uncultured microbial majority. Nat Rev Microbiol. 2020;19:1–16. 10.1038/s41579-020-00458-8 33093661

[bib45] Ling LL , Schneider T, Peoples AJ et al. A new antibiotic kills pathogens without detectable resistance. Nature. 2015;517. 10.1038/nature14098. PMC7414797 25561178

[bib46] Liu H , Xue R, Wang Y et al. FACS-iChip: a high-efficiency iChip system for microbial ‘dark matter’ mining. Mar Life Sci Technol. 2020;3. 10.1007/s42995-020-00067-7. PMC10077213 37073346

[bib47] Liu Q , Liu HC, Zhang JL et al. *Sphingomonas psychrolutea* sp. nov., a psychrotolerant bacterium isolated from glacier ice. Int J Syst Evol Microbiol. 2015;65:2955–9. 26025946 10.1099/ijs.0.000362

[bib48] Liu Y , Ji M, Yu T et al. A genome and gene catalog of glacier microbiomes. Nat Biotechnol. 2022;40:1341–8. 35760913 10.1038/s41587-022-01367-2

[bib49] Lloyd KG , Steen AD, Ladau J et al. Phylogenetically novel uncultured microbial cells dominate Earth microbiomes. Msystems. 2018;3:e00055–18. 30273414 10.1128/mSystems.00055-18 PMC6156271

[bib50] Lutz S , Anesio AM, Edwards A et al. Linking microbial diversity and functionality of Arctic glacial surface habitats. Environ Microbiol. 2017;19:551–65. 27511455 10.1111/1462-2920.13494

[bib51] Lutz S , Bradley JA. 12 Glacial surfaces: functions and biogeography. In: Microbial Life in the Cryosphere and its Feedback on Global Change. Berlin: De Gruyter, 2021, 239–52.

[bib52] Marcolefas E , Leung T, Okshevsky M et al. Culture-dependent bioprospecting of bacterial isolates from the canadian high arctic displaying antibacterial activity. Front Microbiol. 2019;10. 10.3389/fmicb.2019.01836. PMC6696727 31447822

[bib53] Margesin R , Collins T. Microbial ecology of the cryosphere (glacial and permafrost habitats): current knowledge. Appl Microbiol Biotechnol. 2019;103:2537–49. 30719551 10.1007/s00253-019-09631-3 PMC6443599

[bib54] Millar JL , Bagshaw EA, Edwards A et al. Polar cryoconite associated microbiota is dominated by hemispheric specialist genera. Front Microbiol. 2021;12:3318. 10.3389/fmicb.2021.738451 PMC8660574 34899626

[bib55] Mogrovejo DC , Perini L, Gostinčar C et al. Prevalence of antimicrobial resistance and hemolytic phenotypes in culturable Arctic bacteria. Front Microbiol. 2020;11:1–13. 32318045 10.3389/fmicb.2020.00570 PMC7147505

[bib56] Musilova M , Tranter M, Bennett SA et al. Stable microbial community composition on the Greenland Ice Sheet. Front Microbiol. 2015;6:1–10. 25852658 10.3389/fmicb.2015.00193 PMC4367435

[bib57] Nicholes MJ , Williamson CJ, Tranter M et al. Bacterial dynamics in supraglacial habitats of the Greenland Ice Sheet. Front Microbiol. 2019;10. 10.3389/fmicb.2019.01366. PMC6616251 31333595

[bib58] Nurk S , Meleshko D, Korobeynikov A et al. metaSPAdes: a new versatile metagenomic assembler. Genome Res. 2017;27:824–34. 28298430 10.1101/gr.213959.116 PMC5411777

[bib59] Parks DH , Chuvochina M, Waite DW et al. A standardized bacterial taxonomy based on genome phylogeny substantially revises the tree of life. Nat Biotechnol. 2018;36:996. 30148503 10.1038/nbt.4229

[bib60] Perini L , Andrejašič K, Gostinčar C et al. Greenland and Svalbard glaciers host unknown basidiomycetes: the yeast *Camptobasidium arcticum* sp. nov. and the dimorphic *Psychromyces glacialis* gen. and sp. nov. Int J Syst Evol Microbiol. 2021;71:1–17. 10.1099/ijsem.0.004655 PMC8346769 33502296

[bib61] Perini L , Gostinčar C, Anesio AM et al. Darkening of the Greenland Ice Sheet: fungal abundance and diversity are associated with algal bloom. Front Microbiol. 2019;10:1–14. 30949152 10.3389/fmicb.2019.00557 PMC6437116

[bib62] Perini L , Gostinčar C, Likar M et al. Interactions of fungi and algae from the Greenland Ice Sheet. Microb Ecol. 2022;86. 10.1007/s00248-022-02033-5. PMC10293465 35608637

[bib63] Poniecka EA , Bagshaw EA, Sass H et al. Physiological capabilities of cryoconite hole microorganisms. Front Microbiol. 2020;0:1783. 10.3389/fmicb.2020.01783 PMC7412143 32849402

[bib64] Porter C , Morin P, Howat I et al. ArcticDEM. St Paul: Polar Geospatial Center, Digit Inc Imag. 2023. 10.7910/DVN/OHHUKH.

[bib65] Quast C , Pruesse E, Yilmaz P et al. The SILVA ribosomal RNA gene database project: improved data processing and web-based tools. Nucleic Acids Res. 2013;41:D590–6. 23193283 10.1093/nar/gks1219 PMC3531112

[bib66] R Core Team . R: A Language and Environment for Statistical Computing. Vienna: R Foundation for Statistical Computing, 2021.

[bib67] Reuter JA , Spacek DV, Snyder MP. High-throughput sequencing technologies. Mol Cell. 2015;58:586–97. 26000844 10.1016/j.molcel.2015.05.004 PMC4494749

[bib68] Rogers TJ , Buongiorno J, Jessen GL et al. Chemolithoautotroph distributions across the subsurface of a convergent margin. ISME J. 2023;17:140–50. 10.1038/s41396-022-01331-7. 36257972 PMC9751116

[bib69] Šantl-Temkiv T , Gosewinkel U, Starnawski P et al. Aeolian dispersal of bacteria in southwest Greenland: their sources, abundance, diversity and physiological states. FEMS Microbiol Ecol. 2018;94:1–10. 10.1093/femsec/fiy031 29481623

[bib70] Sipes K , Paul R, Fine A et al. Permafrost active layer microbes from Ny Ålesund, Svalbard (79°N) show autotrophic and heterotrophic metabolisms with diverse carbon-degrading enzymes. Front Microbiol. 2022;0:3901. 10.3389/fmicb.2021.757812 PMC8851200 35185810

[bib71] Stamatakis A. RAxML version 8: a tool for phylogenetic analysis and post-analysis of large phylogenies. Bioinformatics. 2014;30:1312–3. 24451623 10.1093/bioinformatics/btu033 PMC3998144

[bib72] Stevens IT , Irvine-Fynn TDL, Edwards A et al. Spatially consistent microbial biomass and future cellular carbon release from melting Northern Hemisphere glacier surfaces. Commun Earth Environ. 2022;3. 10.1038/s43247-022-00609-0.

[bib73] Stibal M , Schostag M, Cameron KA et al. Different bulk and active bacterial communities in cryoconite from the margin and interior of the Greenland Ice Sheet. Environ Microbiol Rep. 2015;7:293–300. 25405749 10.1111/1758-2229.12246

[bib74] Uritskiy GV , DiRuggiero J, Taylor J. MetaWRAP—a flexible pipeline for genome-resolved metagenomic data analysis. Microbiome. 2018;6:158. 30219103 10.1186/s40168-018-0541-1 PMC6138922

[bib44] van Der Linde K , Lim BT, Rondeel JMM et al. Improved bacteriological surveillance of haemodialysis fluids: a comparison between Tryptic soy agar and Reasoner’s 2A media. Nephrol Dial Transplant. 2000;14:2433–37. 10.1093/ndt/14.10.2433 10528669

[bib75] Wehrmann LM , Riedinger N, Brunner B et al. Iron-controlled oxidative sulfur cycling recorded in the distribution and isotopic composition of sulfur species in glacially influenced fjord sediments of west Svalbard. Chem Geol. 2017;466:678–95.

[bib76] Williamson CJ , Cook J, Tedstone A et al. Algal photophysiology drives darkening and melt of the Greenland Ice Sheet. Proc Natl Acad Sci USA. 2020;117:5694–705. 32094168 10.1073/pnas.1918412117 PMC7084142

[bib77] Wu Y-W , Simmons BA, Singer SW. MaxBin 2.0: an automated binning algorithm to recover genomes from multiple metagenomic datasets. Bioinformatics. 2016;32:605–7. 26515820 10.1093/bioinformatics/btv638

