# Supplementary material for: Exploring microbial diversity in Greenland Ice Sheet supraglacial habitats through culturing-dependent and -independent approaches
Source: FEMS Microbiol Ecol. 2023 Oct 3;99(11):fiad119. doi: 10.1093/femsec/fiad119 (PMC10580271; doi:10.1093/femsec/fiad119)
Supplement: fiad119_Supplemental_Files [file fiad119_supplemental_files.zip › Supplementary_captions.docx]

Supplementary data 1: Weather station data

Supplementary data 2: Microbial diversity data: abundance of amplicon/metagenome rRNA, isolates, MAGs including taxonomy and metadata

Supplementary data 3: Alpha diversity indices

Supplementary data 4: Table of obtained isolates

Supplementary data 5: Phylogenetic tree of isolate and metagenome 16S rRNA genes

Supplementary data 6: 16S amplicon sequencing rarefaction curves

Supplementary data 7: 18S amplicon sequencing rarefaction curves
