# Supplementary material for: Exploring microbial diversity in Greenland Ice Sheet supraglacial habitats through culturing-dependent and -independent approaches
Source: FEMS Microbiol Ecol. 2023 Oct 3;99(11):fiad119. doi: 10.1093/femsec/fiad119 (PMC10580271; doi:10.1093/femsec/fiad119)
Supplement: fiad119_Supplemental_Files [file fiad119_supplemental_files.zip › Supplementary_data_4_isolates_table.docx]

| Bacterial Isolates | | |  |  |  |  |  |  |
| --- | --- | --- | --- | --- | --- | --- | --- | --- |
| ID | **Inoculum** | **Method** | **Closest relative, Sanger sequencing** | **% Pairwise Identity** | **Accession** | **Closest relative, WGS extracted 16S rRNA** | **% Pairwise Identity2** | **Accession3** |
| CCC2.4 | cryoconite | culture chamber | *Actimicrobium antarcticum* strain KOPRI 25157 16S ribosomal RNA, partial sequence | 96.50% | NR_118029 | *Actimicrobium antarcticum* strain KOPRI 25157 16S ribosomal RNA, partial sequence | 98.20% | NR_118029 |
| 10I1 | ice | plate | *Cryobacterium breve* strain TMT4-23 16S ribosomal RNA, partial sequence | 98.20% | NR_170453 | *Cryobacterium tepidiphilum* strain NEAU-85 16S ribosomal RNA, partial sequence | 98.00% | NR_164984 |
| 10I5 | ice | plate | *Cryobacterium breve* strain TMT4-23 16S ribosomal RNA, partial sequence | 86.00% | NR_170453 | *Cryobacterium tepidiphilum* strain NEAU-85 16S ribosomal RNA, partial sequence | 98.60% | NR_164984 |
| 10S3 | snow | plate | *Cryobacterium breve* strain TMT4-23 16S ribosomal RNA, partial sequence | 97.40% | NR_170453 | *Cryobacterium tepidiphilum* strain NEAU-85 16S ribosomal RNA, partial sequence | 98.00% | NR_164984 |
| 5B3 | biofilm | plate | *Cryobacterium breve* strain TMT4-23 16S ribosomal RNA, partial sequence | 95.70% | NR_170453 | *Cryobacterium tepidiphilum* strain NEAU-85 16S ribosomal RNA, partial sequence | 98.60% | NR_164984 |
| 5I3 | ice | plate | *Cryobacterium breve* strain TMT4-23 16S ribosomal RNA, partial sequence | 98.10% | NR_170453 | *Cryobacterium tepidiphilum* strain NEAU-85 16S ribosomal RNA, partial sequence | 98.60% | NR_164984 |
| RTC2.1 | cryoconite | plate | *Cryobacterium breve* strain TMT4-23 16S ribosomal RNA, partial sequence | 98.50% | NR_170453 | *Cryobacterium tepidiphilum* strain NEAU-85 16S ribosomal RNA, partial sequence | 98.00% | NR_164984 |
| RTS3 | snow | plate | *Cryobacterium breve* strain TMT4-23 16S ribosomal RNA, partial sequence | 97.00% | NR_170453 | *Cryobacterium tepidiphilum* strain NEAU-85 16S ribosomal RNA, partial sequence | 98.00% | NR_164984 |
| CA11.2 | cryoconite | chip | *Glaciimonas alpina* strain Cr9-12 16S ribosomal RNA, partial sequence | 99.00% | NR_135902 | *Glaciimonas alpina* strain Cr9-12 16S ribosomal RNA, partial sequence | 99.90% | NR_135902 |
| Cout2 | cryoconite | chip | *Glaciimonas alpina* strain Cr9-12 16S ribosomal RNA, partial sequence | 98.60% | NR_135902 | *Glaciimonas alpina* strain Cr9-12 16S ribosomal RNA, partial sequence | 99.90% | NR_135902 |
| Gout2 | cryoconite | chip | *Glaciimonas alpina* strain Cr9-12 16S ribosomal RNA, partial sequence | 98.80% | NR_135902 | *Glaciimonas alpina* strain Cr9-12 16S ribosomal RNA, partial sequence | 99.90% | NR_135902 |
| CCI2.3 | ice | culture chamber | *Actimicrobium antarcticum* strain KOPRI 25157 16S ribosomal RNA, partial sequence | 96.70% | NR_118029 | *Glaciimonas singularis* strain A2-57 16S ribosomal RNA, partial sequence | 97.10% | NR_109670 |
| 10I3 | ice | plate | *Undibacterium seohonense* strain SHS5-24 16S ribosomal RNA, partial sequence | 97.00% | NR_125672 | *Herbaspirillum lusitanum* strain P6-12 16S ribosomal RNA, partial sequence | 96.50% | NR_028859 |
| 5I1 | ice | plate | *Undibacterium seohonense* strain SHS5-24 16S ribosomal RNA, partial sequence | 96.60% | NR_125672 | *Herbaspirillum lusitanum* strain P6-12 16S ribosomal RNA, partial sequence | 96.50% | NR_028859 |
| 5I2 | ice | plate | *Undibacterium jejuense* strain JS4-4 16S ribosomal RNA, partial sequence | 96.60% | NR_125671 | *Herbaspirillum lusitanum* strain P6-12 16S ribosomal RNA, partial sequence | 96.40% | NR_028859 |
| CCC1.1 | cryoconite | culture chamber | *Undibacterium jejuense* strain JS4-4 16S ribosomal RNA, partial sequence | 97.50% | NR_125671 | *Herbaspirillum lusitanum* strain P6-12 16S ribosomal RNA, partial sequence | 96.40% | NR_028859 |
| CCC2.1 | cryoconite | culture chamber | *Undibacterium jejuense* strain JS4-4 16S ribosomal RNA, partial sequence | 96.80% | NR_125671 | *Herbaspirillum lusitanum* strain P6-12 16S ribosomal RNA, partial sequence | 96.30% | NR_028859 |
| CCC3.4 | cryoconite | culture chamber | *Undibacterium jejuense* strain JS4-4 16S ribosomal RNA, partial sequence | 96.30% | NR_125671 | *Herbaspirillum lusitanum* strain P6-12 16S ribosomal RNA, partial sequence | 96.30% | NR_028859 |
| RTI2.1 | ice | plate | Undibacterium pigrum strain CCUG 49009 16S ribosomal RNA, partial sequence | 96.80% | NR_042557 | *Herbaspirillum lusitanum* strain P6-12 16S ribosomal RNA, partial sequence | 96.50% | NR_028859 |
| RTI2.2 | ice | plate | *Undibacterium jejuense* strain JS4-4 16S ribosomal RNA, partial sequence | 95.30% | NR_125671 | *Herbaspirillum lusitanum* strain P6-12 16S ribosomal RNA, partial sequence | 96.50% | NR_028859 |
| RTI4 | ice | plate | *Herbaspirillum lusitanum* strain P6-12 16S ribosomal RNA, partial sequence | 96.00% | NR_028859 | *Herbaspirillum lusitanum* strain P6-12 16S ribosomal RNA, partial sequence | 97.90% | NR_028859 |
| 10C2 | cryoconite | plate | *Cryobacterium breve* strain TMT4-23 16S ribosomal RNA, partial sequence | 98.50% | NR_170453 | *Leifsonia kafniensis* strain KFC-22 16S ribosomal RNA, partial sequence | 98.80% | NR_042669 |
| 10B2 | biofilm | plate | *Mucilaginibacter rigui* strain NBRC 101115 16S ribosomal RNA, partial sequence | 97.90% | NR_113976 | *Mucilaginibacter rigui* strain NBRC 101115 16S ribosomal RNA, partial sequence | 98.80% | NR_113976 |
| 10I4 | ice | plate | *Mucilaginibacter rigui* strain NBRC 101115 16S ribosomal RNA, partial sequence | 98.20% | NR_113976 | *Mucilaginibacter rigui* strain NBRC 101115 16S ribosomal RNA, partial sequence | 98.80% | NR_113976 |
| 5B2 | biofilm | plate | *Mucilaginibacter rigui* strain NBRC 101115 16S ribosomal RNA, partial sequence | 97.50% | NR_113976 | *Mucilaginibacter rigui* strain NBRC 101115 16S ribosomal RNA, partial sequence | 98.80% | NR_113976 |
| 5C4 | cryoconite | plate | *Mucilaginibacter rigui* strain NBRC 101115 16S ribosomal RNA, partial sequence | 97.90% | NR_113976 | *Mucilaginibacter rigui* strain NBRC 101115 16S ribosomal RNA, partial sequence | 98.80% | NR_113976 |
| MH9.3 | ice | chip | *Pseudomonas trivialis* strain P 513/19 16S ribosomal RNA, partial sequence | 98.70% | NR_028987 | *Pseudomonas antarctica* strain CMS 35 16S ribosomal RNA, partial sequence | 99.70% | NR_025586 |
| CCC4.1 | cryoconite | culture chamber | *Pseudomonas fragi* strain NBRC 3458 16S ribosomal RNA, partial sequence | 97.90% | NR_113578 | *Pseudomonas fragi* strain NBRC 3458 16S ribosomal RNA, partial sequence | 100.00% | NR_113578 |
| 10C3 | cryoconite | plate | *Pseudomonas brassicacearum subsp. neoaurantiaca* strain CIP 109457 16S ribosomal RNA, partial sequence | 98.20% | NR_116299 | *Pseudomonas frederiksbergensis* strain DSM 13022 16S ribosomal RNA, partial sequence | 98.70% | NR_117177 |
| 10S4 | snow | plate | *Pseudomonas frederiksbergensis* strain JAJ28 16S ribosomal RNA, partial sequence | 96.60% | NR_028906 | *Pseudomonas frederiksbergensis* strain DSM 13022 16S ribosomal RNA, partial sequence | 99.80% | NR_117177 |
| 5B4 | biofilm | plate | *Pseudomonas brassicacearum subsp. neoaurantiaca* strain CIP 109457 16S ribosomal RNA, partial sequence | 97.50% | NR_116299 | *Pseudomonas frederiksbergensis* strain DSM 13022 16S ribosomal RNA, partial sequence | 98.80% | NR_117177 |
| 5C2 | cryoconite | plate | *Pseudomonas brassicacearum subsp. neoaurantiaca* strain CIP 109457 16S ribosomal RNA, partial sequence | 97.30% | NR_116299 | *Pseudomonas frederiksbergensis* strain DSM 13022 16S ribosomal RNA, partial sequence | 98.80% | NR_117177 |
| 5S1 | snow | plate | *Pseudomonas frederiksbergensis* strain JAJ28 16S ribosomal RNA, partial sequence | 97.70% | NR_028906 | *Pseudomonas frederiksbergensis* strain DSM 13022 16S ribosomal RNA, partial sequence | 99.80% | NR_117177 |
| AH2 | cryoconite | chip | *Pseudomonas lini* strain DLE411J 16S ribosomal RNA, partial sequence | 97.00% | NR_029042 | *Pseudomonas frederiksbergensis* strain DSM 13022 16S ribosomal RNA, partial sequence | 98.70% | NR_117177 |
| CCC2.2 | cryoconite | culture chamber | *Pseudomonas brassicacearum subsp. neoaurantiaca* strain CIP 109457 16S ribosomal RNA, partial sequence | 97.60% | NR_116299 | *Pseudomonas frederiksbergensis* strain DSM 13022 16S ribosomal RNA, partial sequence | 98.80% | NR_117177 |
| CCC4.4 | cryoconite | culture chamber | *Pseudomonas lini* strain DLE411J 16S ribosomal RNA, partial sequence | 96.70% | NR_029042 | *Pseudomonas frederiksbergensis* strain DSM 13022 16S ribosomal RNA, partial sequence | 98.80% | NR_117177 |
| DC1.2 | cryoconite | chip | *Pseudomonas frederiksbergensis* strain JAJ28 16S ribosomal RNA, partial sequence | 97.90% | NR_028906 | *Pseudomonas frederiksbergensis* strain DSM 13022 16S ribosomal RNA, partial sequence | 99.60% | NR_117177 |
| Dout3 | cryoconite | chip | *Pseudomonas frederiksbergensis* strain JAJ28 16S ribosomal RNA, partial sequence | 97.50% | NR_028906 | *Pseudomonas frederiksbergensis* strain DSM 13022 16S ribosomal RNA, partial sequence | 99.50% | NR_117177 |
| MH9.2 | ice | chip | *Pseudomonas brassicacearum subsp. neoaurantiaca* strain CIP 109457 16S ribosomal RNA, partial sequence | 96.70% | NR_116299 | *Pseudomonas frederiksbergensis* strain DSM 13022 16S ribosomal RNA, partial sequence | 98.80% | NR_117177 |
| RTB2 | biofilm | plate | *Pseudomonas brassicacearum subsp. neoaurantiaca* strain CIP 109457 16S ribosomal RNA, partial sequence | 97.10% | NR_116299 | *Pseudomonas frederiksbergensis* strain DSM 13022 16S ribosomal RNA, partial sequence | 98.80% | NR_117177 |
| RTB3 | biofilm | plate | *Pseudomonas frederiksbergensis* strain JAJ28 16S ribosomal RNA, partial sequence | 97.00% | NR_028906 | *Pseudomonas frederiksbergensis* strain DSM 13022 16S ribosomal RNA, partial sequence | 98.70% | NR_117177 |
| RTC3 | cryoconite | plate | *Pseudomonas brassicacearum subsp. neoaurantiaca* strain CIP 109457 16S ribosomal RNA, partial sequence | 97.80% | NR_116299 | *Pseudomonas frederiksbergensis* strain DSM 13022 16S ribosomal RNA, partial sequence | 98.80% | NR_117177 |
| 5S3 | snow | plate | *Pseudomonas lactis* strain DSM 29167 16S ribosomal RNA, partial sequence | 98.50% | NR_156986 | *Pseudomonas gessardii* strain CIP 105469 16S ribosomal RNA, partial sequence | 99.60% | NR_024928 |
| 5S2 | snow | plate | *Pseudomonas azotoformans* strain NBRC 12693 16S ribosomal RNA, partial sequence | 98.90% | NR_113600 | *Pseudomonas lactis* strain DSM 29167 16S ribosomal RNA, partial sequence | 100.00% | NR_156986 |
| CCI1.1 | ice | culture chamber | *Pseudomonas azotoformans* strain NBRC 12693 16S ribosomal RNA, partial sequence | 98.90% | NR_113600 | *Pseudomonas lactis* strain DSM 29167 16S ribosomal RNA, partial sequence | 100.00% | NR_156986 |
| RTS1 | snow | plate | *Pseudomonas azotoformans* strain NBRC 12693 16S ribosomal RNA, partial sequence | 98.30% | NR_113600 | *Pseudomonas lactis* strain DSM 29167 16S ribosomal RNA, partial sequence | 100.00% | NR_156986 |
| RTS2 | snow | plate | *Pseudomonas azotoformans* strain NBRC 12693 16S ribosomal RNA, partial sequence | 98.80% | NR_113600 | *Pseudomonas lactis* strain DSM 29167 16S ribosomal RNA, partial sequence | 100.00% | NR_156986 |
| Bout1 | ice | chip | *Pseudomonas brenneri* strain CFML 97-391 16S ribosomal RNA, partial sequence | 99.30% | NR_025103 | *Pseudomonas migulae* strain CIP 105470 16S ribosomal RNA, partial sequence | 99.40% | NR_024927 |
| CCI1.2 | ice | culture chamber | *Pseudomonas lini* strain DLE411J 16S ribosomal RNA, partial sequence | 96.40% | NR_029042 | *Pseudomonas prosekii* strain AN/28/1 16S ribosomal RNA, partial sequence | 99.00% | NR_132724 |
| 10B1 | biofilm | plate | *Pseudomonas lini* strain DLE411J 16S ribosomal RNA, partial sequence | 96.40% | NR_029042 | *Pseudomonas silesiensis* strain A3 16S ribosomal RNA, complete sequence | 98.90% | NR_156815 |
| 10S5 | snow | plate | *Pseudomonas savastanoi* strain CFBP 1670 16S ribosomal RNA, partial sequence | 97.00% | NR_117822 | *Pseudomonas silesiensis* strain A3 16S ribosomal RNA, complete sequence | 98.90% | NR_156815 |
| 5S4 | snow | plate | *Pseudomonas savastanoi* strain CFBP 1670 16S ribosomal RNA, partial sequence | 97.70% | NR_117822 | *Pseudomonas silesiensis* strain A3 16S ribosomal RNA, complete sequence | 98.90% | NR_156815 |
| AB12 | cryoconite | chip | *Pseudomonas lini* strain DLE411J 16S ribosomal RNA, partial sequence | 95.50% | NR_029042 | *Pseudomonas silesiensis* strain A3 16S ribosomal RNA, complete sequence | 98.90% | NR_156815 |
| AB6 | cryoconite | chip | *Pseudomonas savastanoi* strain CFBP 1670 16S ribosomal RNA, partial sequence | 96.90% | NR_117822 | *Pseudomonas silesiensis* strain A3 16S ribosomal RNA, complete sequence | 98.90% | NR_156815 |
| CCC1.2 | cryoconite | culture chamber | *Pseudomonas lini* strain DLE411J 16S ribosomal RNA, partial sequence | 96.90% | NR_029042 | *Pseudomonas silesiensis* strain A3 16S ribosomal RNA, complete sequence | 98.90% | NR_156815 |
| CCC3.2 | cryoconite | culture chamber | *Pseudomonas lini* strain DLE411J 16S ribosomal RNA, partial sequence | 96.80% | NR_029042 | *Pseudomonas silesiensis* strain A3 16S ribosomal RNA, complete sequence | 98.90% | NR_156815 |
| CCC4.3 | cryoconite | culture chamber | *Pseudomonas lini* strain DLE411J 16S ribosomal RNA, partial sequence | 94.70% | NR_029042 | *Pseudomonas silesiensis* strain A3 16S ribosomal RNA, complete sequence | 98.80% | NR_156815 |
| CCI2.4 | ice | culture chamber | *Pseudomonas lini* strain DLE411J 16S ribosomal RNA, partial sequence | 97.10% | NR_029042 | *Pseudomonas silesiensis* strain A3 16S ribosomal RNA, complete sequence | 98.80% | NR_156815 |
| CCI3.2 | ice | culture chamber | *Pseudomonas weihenstephanensis* strain DSM 29166 16S ribosomal RNA, partial sequence | 99.00% | NR_148764 | *Pseudomonas silesiensis* strain A3 16S ribosomal RNA, complete sequence | 98.80% | NR_156815 |
| CCI4.2 | ice | culture chamber | *Pseudomonas lini* strain DLE411J 16S ribosomal RNA, partial sequence | 96.80% | NR_029042 | *Pseudomonas silesiensis* strain A3 16S ribosomal RNA, complete sequence | 98.70% | NR_156815 |
| MH10 | ice | chip | *Pseudomonas lini* strain DLE411J 16S ribosomal RNA, partial sequence | 95.70% | NR_029042 | *Pseudomonas silesiensis* strain A3 16S ribosomal RNA, complete sequence | 98.80% | NR_156815 |
| MH10out | ice | chip | *Pseudomonas lini* strain DLE411J 16S ribosomal RNA, partial sequence | 97.40% | NR_029042 | *Pseudomonas silesiensis* strain A3 16S ribosomal RNA, complete sequence | 98.70% | NR_156815 |
| RTI1 | ice | plate | *Pseudomonas savastanoi* strain CFBP 1670 16S ribosomal RNA, partial sequence | 97.20% | NR_117822 | *Pseudomonas silesiensis* strain A3 16S ribosomal RNA, complete sequence | 98.90% | NR_156815 |
| RTS4 | snow | plate | *Pseudomonas lini* strain DLE411J 16S ribosomal RNA, partial sequence | 97.40% | NR_029042 | *Pseudomonas silesiensis* strain A3 16S ribosomal RNA, complete sequence | 98.90% | NR_156815 |
| AA4 | cryoconite | chip | *Pseudomonas lini* strain DLE411J 16S ribosomal RNA, partial sequence | 96.80% | NR_029042 | *Pseudomonas silesiensis* strain A3 16S ribosomal RNA, complete sequence | 98.90% | NR_156815 |
| FG1 | cryoconite | chip | *Pseudomonas fildesensis* strain KG01 16S ribosomal RNA, partial sequence | 98.80% | NR_170438 | *Pseudomonas veronii* strain CIP 104663 16S ribosomal RNA, partial sequence | 99.80% | NR_028706 |
| CCC3.1 | cryoconite | culture chamber | *Pseudomonas versuta* strain L10.10 16S ribosomal RNA, partial sequence | 96.50% | NR_149823 | *Pseudomonas versuta* strain L10.10 16S ribosomal RNA, partial sequence | 99.70% | NR_149823 |
| CCI1.4 | ice | culture chamber | *Pseudomonas weihenstephanensis* strain DSM 29166 16S ribosomal RNA, partial sequence | 99.20% | NR_148764 | *Pseudomonas weihenstephanensis* strain DSM 29166 16S ribosomal RNA, partial sequence | 99.70% | NR_148764 |
| CCI3.1 | ice | culture chamber | *Pseudomonas lini* strain DLE411J 16S ribosomal RNA, partial sequence | 97.20% | NR_029042 | *Pseudomonas weihenstephanensis* strain DSM 29166 16S ribosomal RNA, partial sequence | 99.70% | NR_148764 |
| 10B4 | biofilm | plate | *Sphingomonas glacialis* strain C16y 16S ribosomal RNA, partial sequence | 98.80% | NR_117270 | *Sphingomonas glacialis* strain C16y 16S ribosomal RNA, partial sequence | 99.80% | NR_117270 |
| RTI3 | ice | plate | *Subtercola frigoramans* strain DSM 13057 16S ribosomal RNA, partial sequence | 96.40% | NR_115023 | *Subtercola frigoramans* strain K265 16S ribosomal RNA, partial sequence | 97.60% | NR_025075 |
| LG9.2 | cryoconite | chip | *Variovorax boronicumulans* NBRC 103145 16S ribosomal RNA, partial sequence | 97.50% | NR_114214 | *Variovorax ginsengisoli* strain Gsoil 3165 16S ribosomal RNA, partial sequence | 99.00% | NR_112562 |
| RTB1 | biofilm | plate | *Variovorax boronicumulans* NBRC 103145 16S ribosomal RNA, partial sequence | 96.60% | NR_114214 | *Variovorax ginsengisoli* strain Gsoil 3165 16S ribosomal RNA, partial sequence | 99.00% | NR_112562 |
| 10B3.2 | biofilm | plate | *Pseudomonas lini* strain DLE411J 16S ribosomal RNA, partial sequence | 97.40% | NR_029042 |  |  |  |
| 10C1 | cryoconite | plate | *Rhodoferax ferrireducens* T118 16S ribosomal RNA, partial sequence | 97.10% | NR_074760 |  |  |  |
| 5C1 | cryoconite | plate | *Actimicrobium antarcticum* strain KOPRI 25157 16S ribosomal RNA, partial sequence | 96.80% | NR_118029 |  |  |  |
| 5C3 | cryoconite | plate | *Chryseobacterium frigidisoli* strain PB4 16S ribosomal RNA, partial sequence | 96.50% | NR_109469 |  |  |  |
| Cout1 | cryoconite | chip | *Glaciimonas alpina* strain Cr9-12 16S ribosomal RNA, partial sequence | 98.00% | NR_135902 |  |  |  |
| FB7 | cryoconite | chip | *Rhodanobacter umsongensis* strain GR24-2 16S ribosomal RNA, partial sequence | 95.80% | NR_108435 |  |  |  |
| FC1.1 | cryoconite | chip | *Glaciimonas alpina* strain Cr9-12 16S ribosomal RNA, partial sequence | 98.70% | NR_135902 |  |  |  |
| FC3 | cryoconite | chip | *Rhodanobacter umsongensis* strain GR24-2 16S ribosomal RNA, partial sequence | 95.90% | NR_108435 |  |  |  |
| FC4 | cryoconite | chip | *Rhodanobacter koreensis* strain THG-DD7 16S ribosomal RNA, partial sequence | 96.60% | NR_134797 |  |  |  |
| FC6 | cryoconite | chip | *Lacisediminihabitans profunda* strain CHu50b-6-2 16S ribosomal RNA, partial sequence | 95.50% | NR_169497 |  |  |  |
| FF1.1 | cryoconite | chip | *Rhodanobacter umsongensis* strain GR24-2 16S ribosomal RNA, partial sequence | 96.20% | NR_108435 |  |  |  |
| FF8 | cryoconite | chip | *Rhodanobacter ginsengisoli* strain GR17-7 16S ribosomal RNA, partial sequence | 96.70% | NR_044127 |  |  |  |
| IBDplug2 | ice | chip | *Lacisediminihabitans profunda* strain CHu50b-6-2 16S ribosomal RNA, partial sequence | 97.30% | NR_169497 |  |  |  |
| IBDplug3 | ice | chip | *Lacisediminihabitans profunda* strain CHu50b-6-2 16S ribosomal RNA, partial sequence | 96.70% | NR_169497 |  |  |  |
| RTC1 | cryoconite | plate | *Sphingomonas glacialis* strain C16y 16S ribosomal RNA, partial sequence | 99.40% | NR_117270 |  |  |  |

| Yeast isolates | |  |  |  |  |
| --- | --- | --- | --- | --- | --- |
| ID | **Inoculum** | **Method** | **Closest relative, Sanger sequencing** | **% Pairwise Identity** | **Accession** |
| CB1 | cryoconite | chip | *Camptobasidium gelus* CBS 8941 28S rRNA gene, partial sequence; from TYPE material | 91.60% | NG_073579 |
| CCI2.1 | ice | culture chamber | *Camptobasidium gelus* CBS 8941 28S rRNA gene, partial sequence; from TYPE material | 96.00% | NG_073579 |
| CE4 | cryoconite | chip | *Camptobasidium gelus* CBS 8941 28S rRNA gene, partial sequence; from TYPE material | 93.40% | NG_073579 |
| DA6.1 | cryoconite | chip | *Camptobasidium gelus* CBS 8941 28S rRNA gene, partial sequence; from TYPE material | 92.60% | NG_073579 |
| DD3.1 | cryoconite | chip | *Camptobasidium gelus* CBS 8941 28S rRNA gene, partial sequence; from TYPE material | 91.70% | NG_073579 |
| DD3.2 | cryoconite | chip | *Camptobasidium gelus* CBS 8941 28S rRNA gene, partial sequence; from TYPE material | 96.40% | NG_073579 |
| FB2 | cryoconite | chip | *Camptobasidium gelus* CBS 8941 28S rRNA gene, partial sequence; from TYPE material | 94.20% | NG_073579 |
| FC1.3 | cryoconite | chip | *Camptobasidium gelus* CBS 8941 28S rRNA gene, partial sequence; from TYPE material | 96.40% | NG_073579 |
| FG6 | cryoconite | chip | *Camptobasidium gelus* CBS 8941 28S rRNA gene, partial sequence; from TYPE material | 93.20% | NG_073579 |
| LA6.1 | cryoconite | chip | *Camptobasidium gelus* CBS 8941 28S rRNA gene, partial sequence; from TYPE material | 97.20% | NG_073579 |
| LD1 | cryoconite | chip | *Camptobasidium gelus* CBS 8941 28S rRNA gene, partial sequence; from TYPE material | 92.20% | NG_073579 |
| LD10.2 | cryoconite | chip | *Camptobasidium gelus* CBS 8941 28S rRNA gene, partial sequence; from TYPE material | 95.50% | NG_073579 |
| LD2 | cryoconite | chip | *Camptobasidium gelus* CBS 8941 28S rRNA gene, partial sequence; from TYPE material | 89.90% | NG_073579 |
| LD9.2 | cryoconite | chip | *Camptobasidium gelus* CBS 8941 28S rRNA gene, partial sequence; from TYPE material | 94.10% | NG_073579 |
| LE10.2 | cryoconite | chip | *Camptobasidium gelus* CBS 8941 28S rRNA gene, partial sequence; from TYPE material | 95.60% | NG_073579 |
| LF3.1 | cryoconite | chip | *Camptobasidium gelus* CBS 8941 28S rRNA gene, partial sequence; from TYPE material | 96.80% | NG_073579 |
| Loutside1 | cryoconite | chip | *Camptobasidium gelus* CBS 8941 28S rRNA gene, partial sequence; from TYPE material | 96.50% | NG_073579 |
| OD4 | cryoconite | chip | *Camptobasidium gelus* CBS 8941 28S rRNA gene, partial sequence; from TYPE material | 92.30% | NG_073579 |
| OE1 | cryoconite | chip | *Camptobasidium gelus* CBS 8941 28S rRNA gene, partial sequence; from TYPE material | 95.80% | NG_073579 |
| OE12 | cryoconite | chip | *Camptobasidium gelus* CBS 8941 28S rRNA gene, partial sequence; from TYPE material | 94.80% | NG_073579.1 |
| OF1 | cryoconite | chip | *Camptobasidium gelus* CBS 8941 28S rRNA gene, partial sequence; from TYPE material | 96.70% | NG_073579 |
| OG3.1 | cryoconite | chip | *Camptobasidium gelus* CBS 8941 28S rRNA gene, partial sequence; from TYPE material | 98.40% | NG_073579 |
| OG3.2 | cryoconite | chip | *Camptobasidium gelus* CBS 8941 28S rRNA gene, partial sequence; from TYPE material | 93.10% | NG_073579 |
| Boutside2 | ice | chip | *Dothiora europaea* CBS 739.71 28S rRNA gene, partial sequence; from TYPE material | 99.60% | NG_064093.1 |
| B1 | ice | plate | *Mrakia gelida* CBS 5272 28S rRNA gene, partial sequence; from TYPE material | 99.00% | NG_070556 |
| B5 | ice | plate | *Mrakia gelida* CBS 5272 28S rRNA gene, partial sequence; from TYPE material | 99.20% | NG_070556 |
| CB8 | cryoconite | chip | *Mrakia gelida* CBS 5272 28S rRNA gene, partial sequence; from TYPE material | 96.40% | NG_070556 |
| CC10 | cryoconite | chip | *Mrakia gelida* CBS 5272 28S rRNA gene, partial sequence; from TYPE material | 98.00% | NG_070556 |
| CC9 | cryoconite | chip | *Mrakia gelida* CBS 5272 28S rRNA gene, partial sequence; from TYPE material | 98.30% | NG_070556 |
| CCC2.3 | cryoconite | culture chamber | *Mrakia gelida* CBS 5272 28S rRNA gene, partial sequence; from TYPE material | 97.50% | NG_070556 |
| CD5 | cryoconite | chip | *Mrakia gelida* CBS 5272 28S rRNA gene, partial sequence; from TYPE material | 99.30% | NG_070556 |
| CE6 | cryoconite | chip | *Mrakia gelida* CBS 5272 28S rRNA gene, partial sequence; from TYPE material | 97.10% | NG_070556 |
| CF4 | cryoconite | chip | *Mrakia gelida* CBS 5272 28S rRNA gene, partial sequence; from TYPE material | 98.30% | NG_070556 |
| CF9 | cryoconite | chip | *Mrakia gelida* CBS 5272 28S rRNA gene, partial sequence; from TYPE material | 96.90% | NG_070556 |
| CH4 | cryoconite | chip | *Mrakia gelida* CBS 5272 28S rRNA gene, partial sequence; from TYPE material | 99.30% | NG_070556 |
| DA8 | cryoconite | chip | *Mrakia gelida* CBS 5272 28S rRNA gene, partial sequence; from TYPE material | 99.30% | NG_070556 |
| DC1.1 | cryoconite | chip | *Mrakia gelida* CBS 5272 28S rRNA gene, partial sequence; from TYPE material | 98.60% | NG_070556 |
| DE2 | cryoconite | chip | *Mrakia gelida* CBS 5272 28S rRNA gene, partial sequence; from TYPE material | 98.50% | NG_070556 |
| DH1 | cryoconite | chip | *Mrakia gelida* CBS 5272 28S rRNA gene, partial sequence; from TYPE material | 98.40% | NG_070556 |
| DH2 | cryoconite | chip | *Mrakia gelida* CBS 5272 28S rRNA gene, partial sequence; from TYPE material | 99.30% | NG_070556 |
| DH3 | cryoconite | chip | *Mrakia gelida* CBS 5272 28S rRNA gene, partial sequence; from TYPE material | 99.30% | NG_070556 |
| FA7 | cryoconite | chip | *Mrakia gelida* CBS 5272 28S rRNA gene, partial sequence; from TYPE material | 98.20% | NG_070556 |
| FB1 | cryoconite | chip | *Mrakia gelida* CBS 5272 28S rRNA gene, partial sequence; from TYPE material | 99.00% | NG_070556 |
| FB6 | cryoconite | chip | *Mrakia gelida* CBS 5272 28S rRNA gene, partial sequence; from TYPE material | 99.30% | NG_070556 |
| FB8 | cryoconite | chip | *Mrakia gelida* CBS 5272 28S rRNA gene, partial sequence; from TYPE material | 99.30% | NG_070556 |
| FC1.4 | cryoconite | chip | *Mrakia gelida* CBS 5272 28S rRNA gene, partial sequence; from TYPE material | 99.50% | NG_070556 |
| FC11 | cryoconite | chip | *Mrakia gelida* CBS 5272 28S rRNA gene, partial sequence; from TYPE material | 98.80% | NG_070556 |
| FD4 | cryoconite | chip | *Mrakia gelida* CBS 5272 28S rRNA gene, partial sequence; from TYPE material | 99.20% | NG_070556 |
| FE7 | cryoconite | chip | *Mrakia gelida* CBS 5272 28S rRNA gene, partial sequence; from TYPE material | 99.20% | NG_070556 |
| FG3 | cryoconite | chip | *Mrakia gelida* CBS 5272 28S rRNA gene, partial sequence; from TYPE material | 99.70% | NG_070556 |
| FH1 | cryoconite | chip | *Mrakia gelida* CBS 5272 28S rRNA gene, partial sequence; from TYPE material | 99.00% | NG_070556 |
| GA6.2 | cryoconite | chip | *Mrakia gelida* CBS 5272 28S rRNA gene, partial sequence; from TYPE material | 99.00% | NG_070556 |
| GD5 | cryoconite | chip | *Mrakia gelida* CBS 5272 28S rRNA gene, partial sequence; from TYPE material | 97.30% | NG_070556 |
| GD6 | cryoconite | chip | *Mrakia gelida* CBS 5272 28S rRNA gene, partial sequence; from TYPE material | 99.30% | NG_070556 |
| GE4 | cryoconite | chip | *Mrakia gelida* CBS 5272 28S rRNA gene, partial sequence; from TYPE material | 99.30% | NG_070556 |
| GE5 | cryoconite | chip | *Mrakia gelida* CBS 5272 28S rRNA gene, partial sequence; from TYPE material | 98.10% | NG_070556 |
| GE6 | cryoconite | chip | *Mrakia gelida* CBS 5272 28S rRNA gene, partial sequence; from TYPE material | 99.50% | NG_070556 |
| GF4 | cryoconite | chip | *Mrakia gelida* CBS 5272 28S rRNA gene, partial sequence; from TYPE material | 98.80% | NG_070556 |
| GF6 | cryoconite | chip | *Mrakia gelida* CBS 5272 28S rRNA gene, partial sequence; from TYPE material | 97.90% | NG_070556 |
| Houtside1 | ice | chip | *Mrakia gelida* CBS 5272 28S rRNA gene, partial sequence; from TYPE material | 97.40% | NG_070556.1 |
| LA6.2 | cryoconite | chip | *Mrakia gelida* CBS 5272 28S rRNA gene, partial sequence; from TYPE material | 98.30% | NG_070556 |
| LC9.1 | cryoconite | chip | *Mrakia gelida* CBS 5272 28S rRNA gene, partial sequence; from TYPE material | 97.10% | NG_070556 |
| LD10.1 | cryoconite | chip | *Mrakia gelida* CBS 5272 28S rRNA gene, partial sequence; from TYPE material | 97.80% | NG_070556 |
| LD5.1 | cryoconite | chip | *Mrakia gelida* CBS 5272 28S rRNA gene, partial sequence; from TYPE material | 99.30% | NG_070556 |
| LD9.1 | cryoconite | chip | *Mrakia gelida* CBS 5272 28S rRNA gene, partial sequence; from TYPE material | 99.50% | NG_070556 |
| LD9.3 | cryoconite | chip | *Mrakia gelida* CBS 5272 28S rRNA gene, partial sequence; from TYPE material | 99.50% | NG_070556 |
| LE10.1 | cryoconite | chip | *Mrakia gelida* CBS 5272 28S rRNA gene, partial sequence; from TYPE material | 97.50% | NG_070556 |
| LF9.1 | cryoconite | chip | *Mrakia gelida* CBS 5272 28S rRNA gene, partial sequence; from TYPE material | 99.70% | NG_070556 |
| LG9.4 | cryoconite | chip | *Mrakia gelida* CBS 5272 28S rRNA gene, partial sequence; from TYPE material | 98.00% | NG_070556 |
| OG3.3 | cryoconite | chip | *Mrakia gelida* CBS 5272 28S rRNA gene, partial sequence; from TYPE material | 99.10% | NG_070556 |
| DC6 | cryoconite | chip | *Phenoliferia psychrophenolica* CBS 10438 28S rRNA gene, partial sequence; from TYPE material | 93.30% | NG_066183.1 |
| CCI3.3 | ice | culture chamber | *Piskurozyma fildesensis* CBS 12705 28S rRNA gene, partial sequence; from TYPE material | 100.00% | NG_073593.1 |
