# Supplementary figures and images for: Exploring microbial diversity in Greenland Ice Sheet supraglacial habitats through culturing-dependent and -independent approaches
Source: FEMS Microbiol Ecol. 2023 Oct 3;99(11):fiad119. doi: 10.1093/femsec/fiad119 (PMC10580271; doi:10.1093/femsec/fiad119)

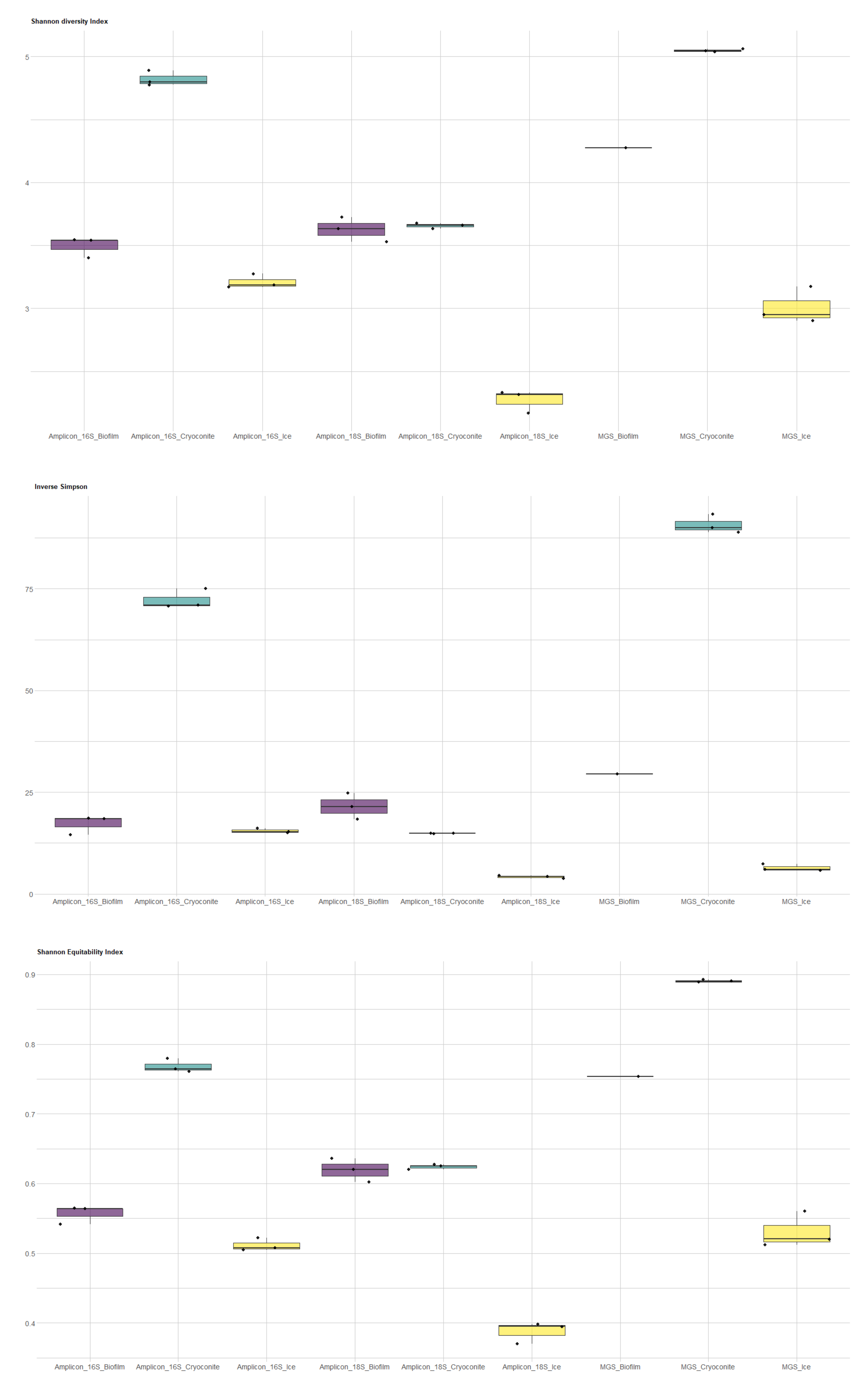

Supplement: fiad119_Supplemental_Files [file fiad119_supplemental_files.zip › Supplementary_data_3_alphadiv.png]

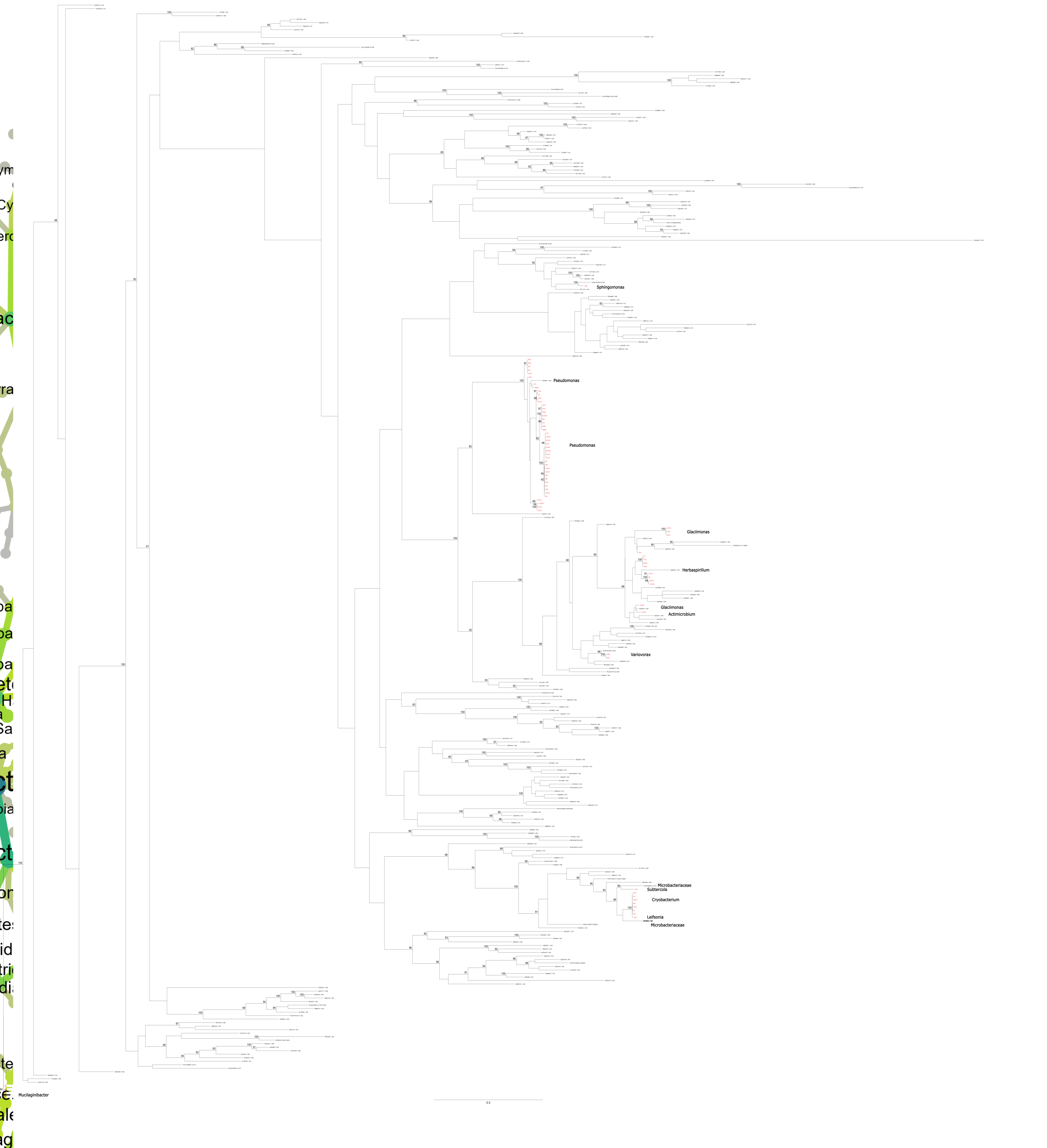

Supplement: fiad119_Supplemental_Files [file fiad119_supplemental_files.zip › Supplementary_data_5_16S_phylo_tree.png]

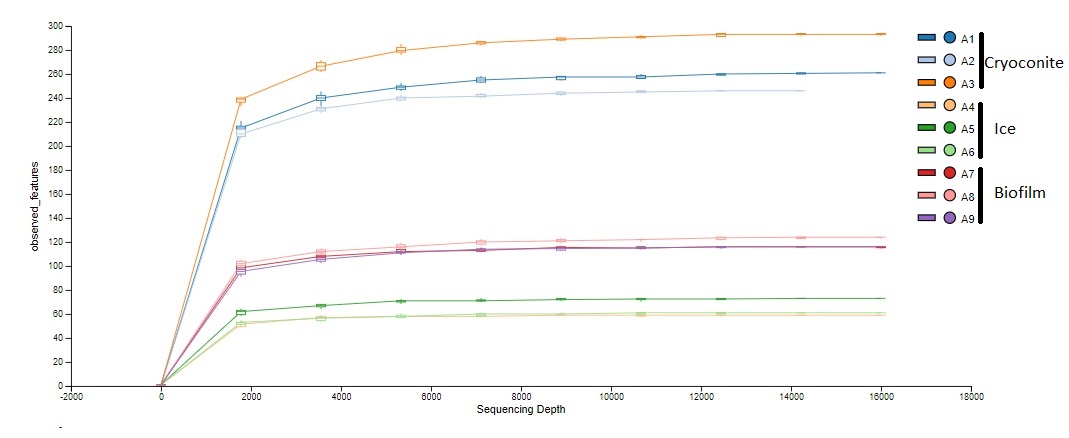

Supplement: fiad119_Supplemental_Files [file fiad119_supplemental_files.zip › Supplementary_data_6_16S_rarefaction.jpg]

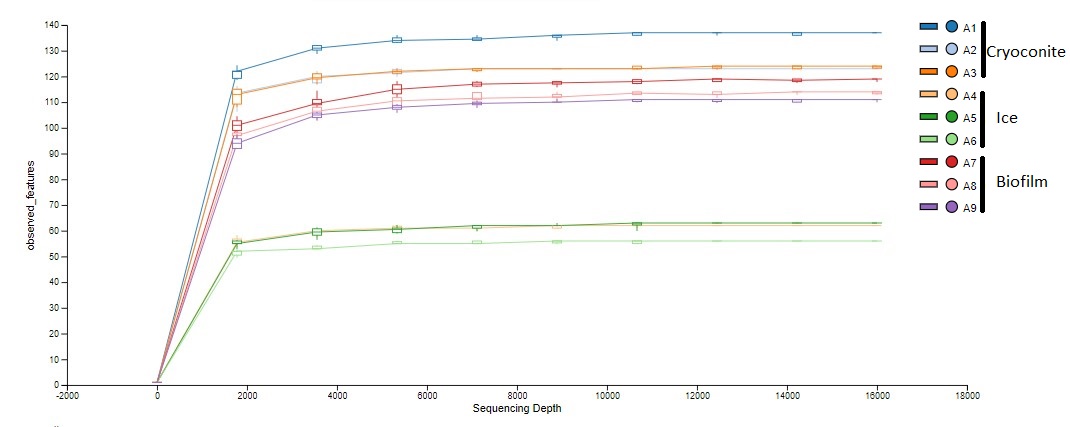

Supplement: fiad119_Supplemental_Files [file fiad119_supplemental_files.zip › Supplementary_data_7_18S_rarefaction.jpg]
